# Spiking Neural P Systems with Neuron Division and Dissolution

**DOI:** 10.1371/journal.pone.0162882

**Published:** 2016-09-14

**Authors:** Yuzhen Zhao, Xiyu Liu, Wenping Wang

**Affiliations:** School of Management Science and Engineering, Shandong Normal University, Jinan, China; University of the West of England, UNITED KINGDOM

## Abstract

Spiking neural P systems are a new candidate in spiking neural network models. By using neuron division and budding, such systems can generate/produce exponential working space in linear computational steps, thus provide a way to solve computational hard problems in feasible (linear or polynomial) time with a “time-space trade-off” strategy. In this work, a new mechanism called neuron dissolution is introduced, by which redundant neurons produced during the computation can be removed. As applications, uniform solutions to two NP-hard problems: SAT problem and Subset Sum problem are constructed in linear time, working in a deterministic way. The neuron dissolution strategy is used to eliminate invalid solutions, and all answers to these two problems are encoded as indices of output neurons. Our results improve the one obtained in *Science China Information Sciences*, 2011, 1596-1607 by Pan et al.

## Introduction

Spiking neural P systems (in short, SN P systems) are a class of bio-inspired parallel computing models, initiated by Ionescu, Păun and Yokomori in 2006 [1], which are inspired from information processing strategy and communication strategy between neurons. A SN P system is constructed by a group of neurons (a class of cells with only one membrane) communicating by sending signals (spikes, represented by object *a*) to neighboring neurons through synapses. Each neuron has a certain number of spikes and rules. Spikes can evolve through application of rules. Since SN P systems were proposed, they become a rapid developing area of membrane computing [2–15].

Researchers pay close attention to computational efficiency of SN P systems, especially the judgement whether NP-complete problems have solutions or not in feasible time [16–26]. If a NP-complete problem has a solution, the output neuron outputs a spike; otherwise, the output neuron outputs nothing. However, we need to find out the solutions in many situations. For instance, the register allocation problem is an application of SAT problem. This problem aims to build a mapping relationship between the virtual registers and the physical registers, and realize the rational utilization of physical register resources. In this case, we need to judge whether a good solution exists, while searching the solution by distributing the physical register resources according to the solution. In applications, many problems can be transformed into graph coloring problems, which is equivalent to SAT problems. To solve these problems, exact solutions are also essential.

For this purpose, neuron dissolution, which is a basic biological phenomenon aiming to remove unnecessary neurons, is introduced into SN P systems [27, 28], and a new class of SN P systems, SN P systems with neuron division and dissolution (DDSN P systems, for short) is proposed in this work. In DDSN P systems, division rules can generate exponent work space (in terms of neurons) which can be used to enumerate all possible results (one result is contained in one neuron), and dissolution rules can dissolve redundant neurons which can be used to remove wrong results. Neurons which represent all possible results are set as output neurons, and these output neurons with invalid results are dissolved in computational process. When the computation halts, the remaining output neurons show all right results. Uniform solutions to SAT problem and Subset Sum problem, which work in a deterministic way, are constructed as examples in this work.

The contributions of this work focus on the following three aspects. 1. The computational space efficiency is improved. If these redundant neurons are reserved, they will occupy huge computational resources such as storage. The dissolution rule can reduce the computational space needed and improve the computational space efficiency. 2. The system structure is clearer. If the redundant neurons are reserved, the SN P system will become complicated, and the useful neurons are not highlighted enough. By introducing the neuron dissolution mechanism, redundant neurons are dissolved immediately, and each of the remaining neuron has its function. 3. Exact solutions to NP-complete problems can be obtained in linear time. Invalid solutions are eliminated during the computational process by neuron dissolution, and all solutions are encoded as indices of specific output neurons at halting, which can provide more valuable information for applications. Uniform solutions to SAT and Subset Sum problems are solved as examples.

The paper is organized as follows. Section 1 defines the SN P systems with neuron division and dissolution. Uniform solutions to SAT and Subset Sum problems in linear time using the proposed SN P systems with neuron division and dissolution are presented in section 2 and section 3. Conclusions are given in section 4.

## 1 SN P Systems with Neuron Division and Dissolution

### 1.1 Background

Biological systems, such as cells, tissues, and human brains, have deep computational intelligence. Biologically inspired computing, or bio-inspired computing in short, focuses on regenerating computing architecture from biological systems to construct computing models and algorithms. Membrane computing is a novel research branch of bio-inspired computing, initiated by Gh. Păun in 2002, which seeks to discover new computational models from the study of biological cells, particularly of the biological membranes [29, 30]. The obtained models are distributed and parallel bio-inspired computing devices, usually called P systems. There are three mainly investigated P systems, cell-like P systems, tissue P systems, and neural-like P systems (also known as spiking neural P systems). P systems, known as powerful computing models, are able to do what Turing machine can do, even solving computational hard problems [31–37].

SN P systems, as a new branch of membrane computing, are a shift from the cell-like architecture to the neural-like architecture. The topological structure of SN P systems is a directed graph: neurons are placed in the vertices of the graph, and synapses act as edges. Each neuron can have a certain number of objects *a* (spikes) and a certain number of *firing* rules and *forgetting* rules. Through a firing rule, a neuron can send information to other neurons by emitting spikes to these neurons. Through a forgetting rule, a certain amount of spikes can be removed from a neuron. Both the firing rules and the forgetting rules have conditions of applied. If the number of spikes in a neuron is contained within in the number set of spikes determined by a regular expression, a rule can have the possibility to be applied. At each time step, one rule is non-deterministically chosen to be applied in each neuron. That is to say, rules are applied in a sequential manner from the view of the neuron, and in parallel from the view of the whole system.

Pan et al. introduced a novel idea to solve SAT problem in polynomial time by using neuron division and budding [25], which uses the neuron division and budding rules to generate more neurons according to the need in the computational process. Wang et al. proved that SN P systems with neuron division, not using neuron budding, can also solve SAT problem in polynomial time [26]. The biological motivation of neuron division and budding comes from the neural stem cells division. The neural stem cells have the ability to proliferate and differentiate into neurons, astrocytes and oligodendrocytes, therefore, they can supply massive tissue cells. In these SN P systems, neuron division rules and neuron budding rules are used to regenerate the above biological phenomena.

In neurons, there is another biological phenomenon called neuron apoptosis, which has a close relationship with neuron division and budding. Neuron apoptosis is a programmed neuron death controlled by a series of activities controlled by genes, such as the activation, expression and regulation of genes. It is not a self damage phenomenon under the pathological condition, but a actively death process. When unnecessary neurons or abnormal neurons occur in the process of neuron development or under the influence of some factors, neuron apoptosis can remove these neurons in multicellular organism to maintain a stable internal environment and to adapt to the environment better. It plays an important role in the evolution of the organism, the stability of internal environment and the development of multiple systems.

For the above biological phenomena, the neuron apoptosis mechanism is introduced into SN P systems, and neuron dissolution rule is designed. In this way, redundant neurons can be eliminated immediately.

### 1.2 System description

A SN P system with neuron division and dissolution of degree *m* is a construct of the form

$$\Pi =(O,H,syn,n_{1},n_{2},\ldots ,n_{m},R,in,out),$$

where:

1. *O* = {*a*} represents the *singleton alphabet* where *a* is the spike;
2. *H* represents the set of *labels* for neurons;
3. *syn* ⊆ *H* × *H* represents a *synapse dictionary* (for each 1 ≤ *i* ≤ *m*, (*i*, *i*) ∉ *syn*);
4. *n*_*i*_ ≥ 0 represents *the spike numbers* in neuron *σ*_*i*_ in the initial state (1 ≤ *i* ≤ *m*);
5. *R* represents the set of all *developmental rules* of the following four forms
  - *firing rule* [*E*/*a*^*c*^ → *a*^*p*^; *d*]_*i*_, where, *i* ∈ *H*, *E* is a regular expression over *a*, *c* ≥ 1, *p* ≥ 1, *c* ≥ *p*, *d* ≥ 0. If *E* = *a*^*c*^, the firing rule is simply written as [*a*^*c*^ → *a*^*p*^; *d*]_*i*_. If *d* = 0, the firing rule is simply written as [*E*/*a*^*c*^ → *a*^*p*^]_*i*_. If *E* = *a*^*c*^ and *d* = 0, the firing rule is simply written as [*a*^*c*^ → *a*^*p*^]_*i*_;
  - *forgetting rule* [*E*/*a*^*s*^ → λ]_*i*_, where, *i* ∈ *H*, *E* is a regular expression over *a*, *s* ≥ 1. If *E* = *a*^*s*^, the forgetting rule is simply written as [*a*^*s*^ → λ]_*i*_;
  - *neuron division rule* [*E*]_*i*_ → [ ]_*j*_ || [ ]_*k*_, where, *i*, *j*, *k* ∈ *H*, *E* is a regular expression over *a*;
  - *neuron dissolution rule* [*E*]_*i*_ → *δ*, where, *i* ∈ *H*, *E* is a regular expression over *a*, object *δ* represents that neuron *σ*_*i*_ is dissolved;
6. *in*, *out* ⊆ *H* represent *the input and output neurons* of Π, respectively.

The synapse dictionary *syn* shows the initial structure of the system and guides how to establish new synapses when new neurons are established.

If neuron *σ*_*i*_ has *h* spikes, and *a*^*h*^ ∈ *L*(*E*), *h* ≥ *c*, the firing rule [*E*/*a*^*c*^ → *a*^*p*^; *d*]_*i*_ can be applied. *c* spikes are consumed (*h* − *c* spikes remain in neuron *σ*_*i*_.), and *p* spikes are emitted after *d* time units (steps). If *d* = 0, *p* spikes are emitted immediately; if *d* = 1, *p* spikes are emitted at the next step; if this firing rule is applied at step *t* and *d* ≥ 1, *p* spikes are emitted at step *t* + *d*. Neuron *σ*_*i*_ is closed at steps *t*, *t* + 1, *t* + 2, …, *t* + *d* − 1, which means no rule will be applied and no spike will be received in this period. At step *t* + *d*, neuron *σ*_*i*_ becomes open again, and can receive new spikes. Once these *p* spikes are emitted from neuron *σ*_*i*_, they reach each neuron *σ*_*j*_ which has a synapse going from neuron *σ*_*i*_ to neuron *σ*_*j*_ and is open. The spikes sent to a closed neuron are *lost*.

If neuron *σ*_*i*_ has *h* spikes, and *a*^*h*^ ∈ *L*(*E*), *h* ≥ *s*, the forgetting rule [*E*/*a*^*s*^ → λ]_*i*_ can be applied. *s* spikes are consumed immediately.

If (1). neuron *σ*_*i*_ has *h* spikes, and *a*^*h*^ ∈ *L*(*E*), and (2). no synapse (*i*, *j*), (*j*, *i*), (*i*, *k*), (*k*, *i*) exists in the system, the neuron division rule [*E*]_*i*_ → [ ]_*j*_ || [ ]_*k*_ can be applied. All *h* spikes in neuron *σ*_*i*_ are consumed and neuron *σ*_*i*_ is divided into two neurons *σ*_*j*_ and *σ*_*k*_. No spike is in neurons *σ*_*j*_ and *σ*_*k*_ at this moment. The labels of the two generated neurons can be different or the same, and the labels of the two generated neurons can be different from or the same with the label of their father neuron *σ*_*i*_, too. The new generated neurons inherit the synapses of their father neuron *σ*_*i*_. That is to say, if there is a synapse (*i*, *g*) going from neuron *σ*_*i*_ to neuron *σ*_*g*_, two synapses (*j*, *g*) and (*k*, *g*) are established after the division rule is applied; if there is a synapse (*g*, *i*) going from neuron *σ*_*g*_ to neuron *σ*_*i*_, two synapses (*g*, *j*) and (*g*, *k*) are established after the division rule is applied. In addition to inheritance of synapses, new generated neurons also have synapses provided by the synapse dictionary *syn*. Synapses not existing in the synapse dictionary *syn* may appear because of inheritance of synapses. The condition (2) avoids the situation that the start and the end of a synapse are the same neuron. For example, if synapse (*i*, *j*) exists in the system, synapses (*j*, *j*), (*k*, *j*) will appear which is not permitted.

**Fig 1:**
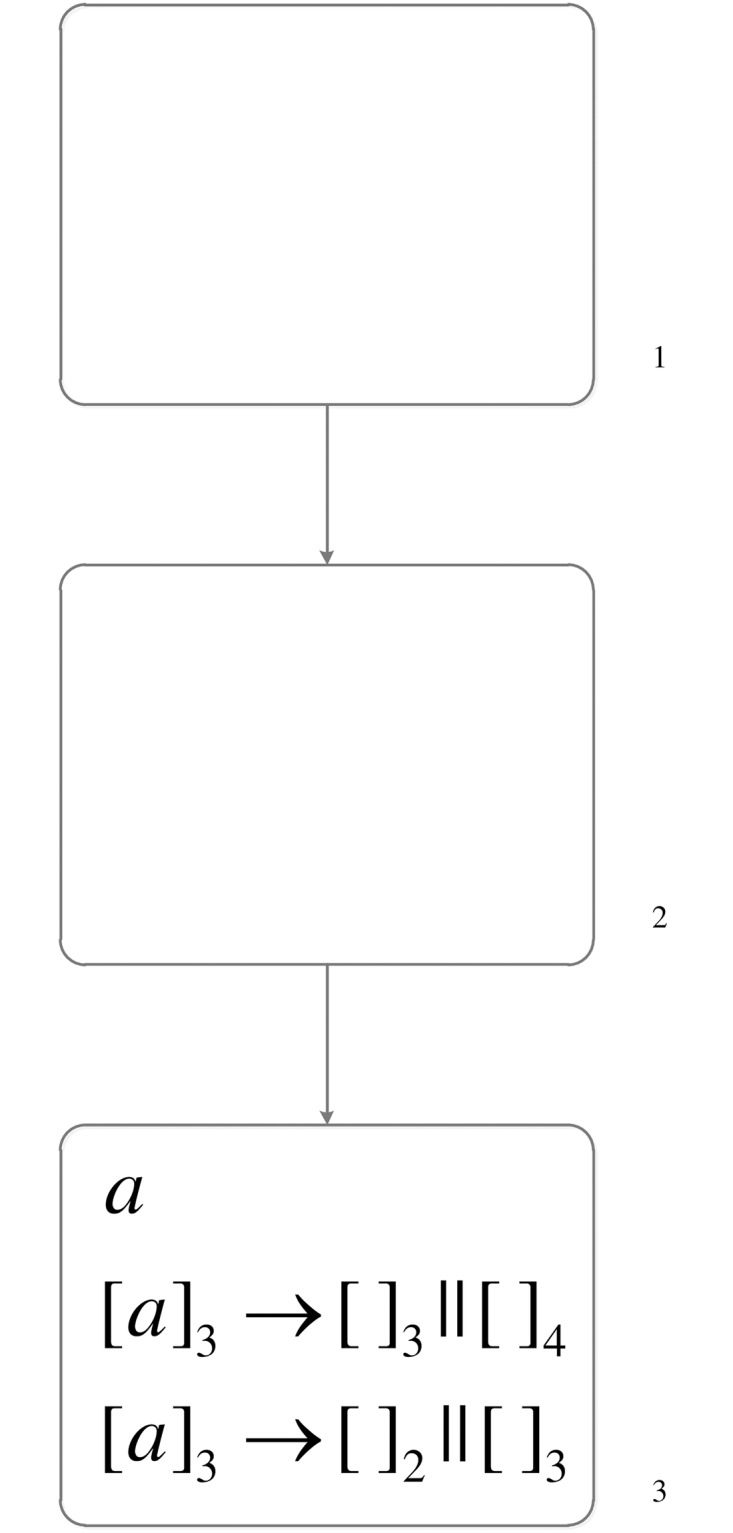
The SN P system before executing division rule.

**Fig 2:**
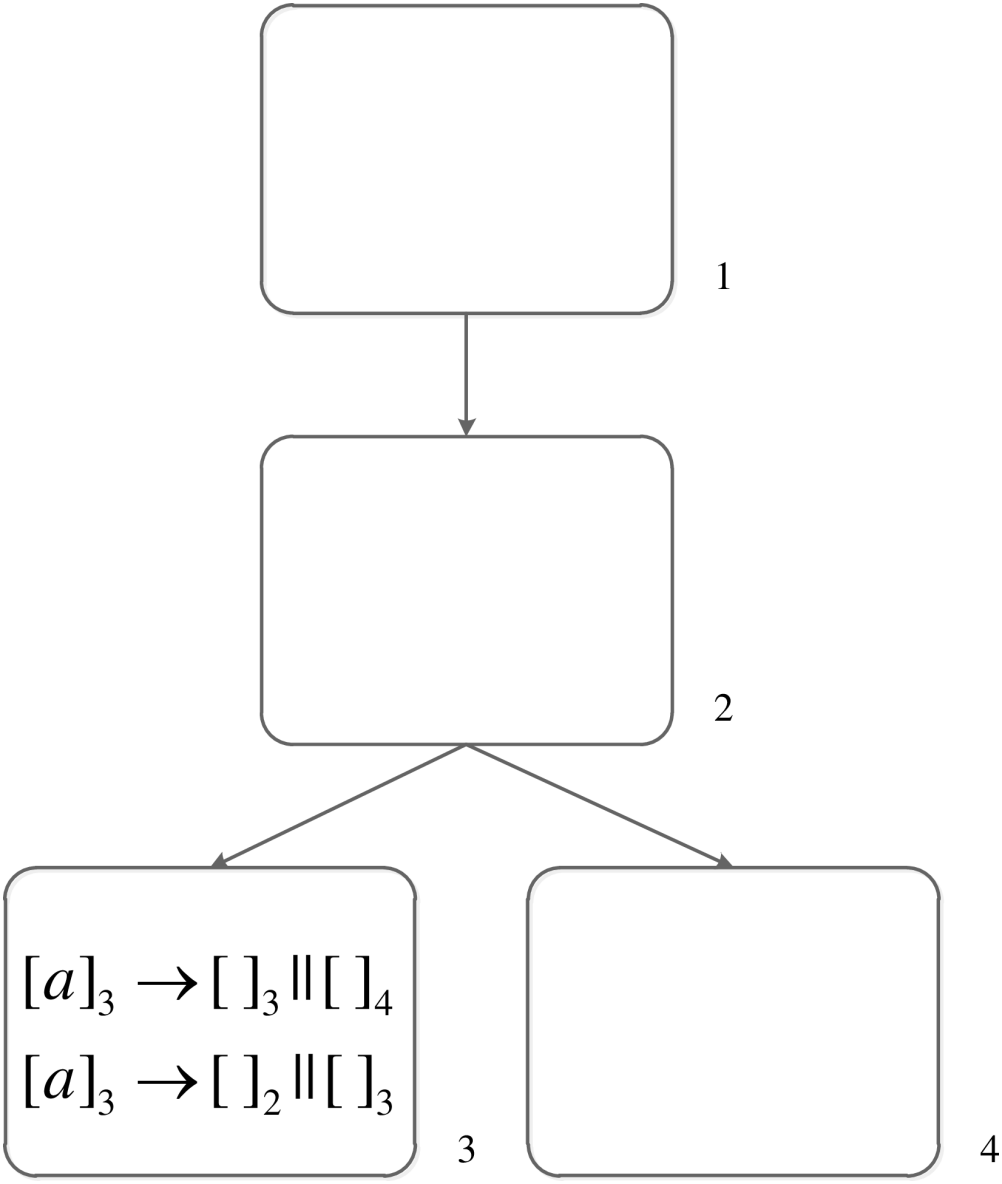
The SN P system after executing division rule.

A simple example shown in Fig 1 is used to show how division rules are applied. One spike and two division rules are in neuron *σ*_3_. Considering the two conditions mentioned in the above paragraph, 1). Neuron *σ*_3_ has one spike *a* and the regular expressions of both two division rules are exactly {*a*}, where *a* ∈ {*a*}. Therefore, both of these two division rules meet the condition (1). 2). For rule [*a*]_3_ → [ ]_2_ || [ ]_3_, the label 3 of the father neuron *σ*_3_ corresponds to *i* in the normalization rule, and the label 2 and 3 of the two new neurons *σ*_2_ and *σ*_3_ corresponds to *j* and *k* in the normalization rule. Synapses (*i*, *j*), (*j*, *i*), (*i*, *k*), (*k*, *i*) cannot exist in the system. That is to say, (3, 2), (2, 3), (3, 3), (3, 3) cannot exist in this system. However, a synapse (2, 3) is in this system, therefore rule [*a*]_3_ → [ ]_2_ || [ ]_3_ cannot be applied. Only rule [*a*]_3_ → [ ]_3_ || [ ]_4_ meet the two conditions. The spike *a* in neuron *σ*_3_ is consumed, neuron *σ*_3_ is divided into two neurons *σ*_3_ and *σ*_4_, and two synapses (2, 3), (2, 4) going from neuron *σ*_2_ to these two new neurons are established because there is a synapse going from neuron *σ*_2_ to the father neuron *σ*_3_ of the two new neurons (the inheritance of synapses). Because rules in this system are related to the labels of neurons, the new neuron *σ*_3_ contains these two rules. The system is changed to Fig 2 after applying rule [*a*]_3_ → [ ]_3_ || [ ]_4_.

If neuron *σ*_*i*_ has *h* spikes, and *a*^*h*^ ∈ *L*(*E*), the neuron dissolution rule [*E*]_*i*_ → *δ* can be applied. All *h* spikes in neuron *σ*_*i*_ are consumed and neuron *σ*_*i*_ is dissolved. All synapses going from/to neuron *σ*_*i*_ are dissolved, too.

**Fig 3:**
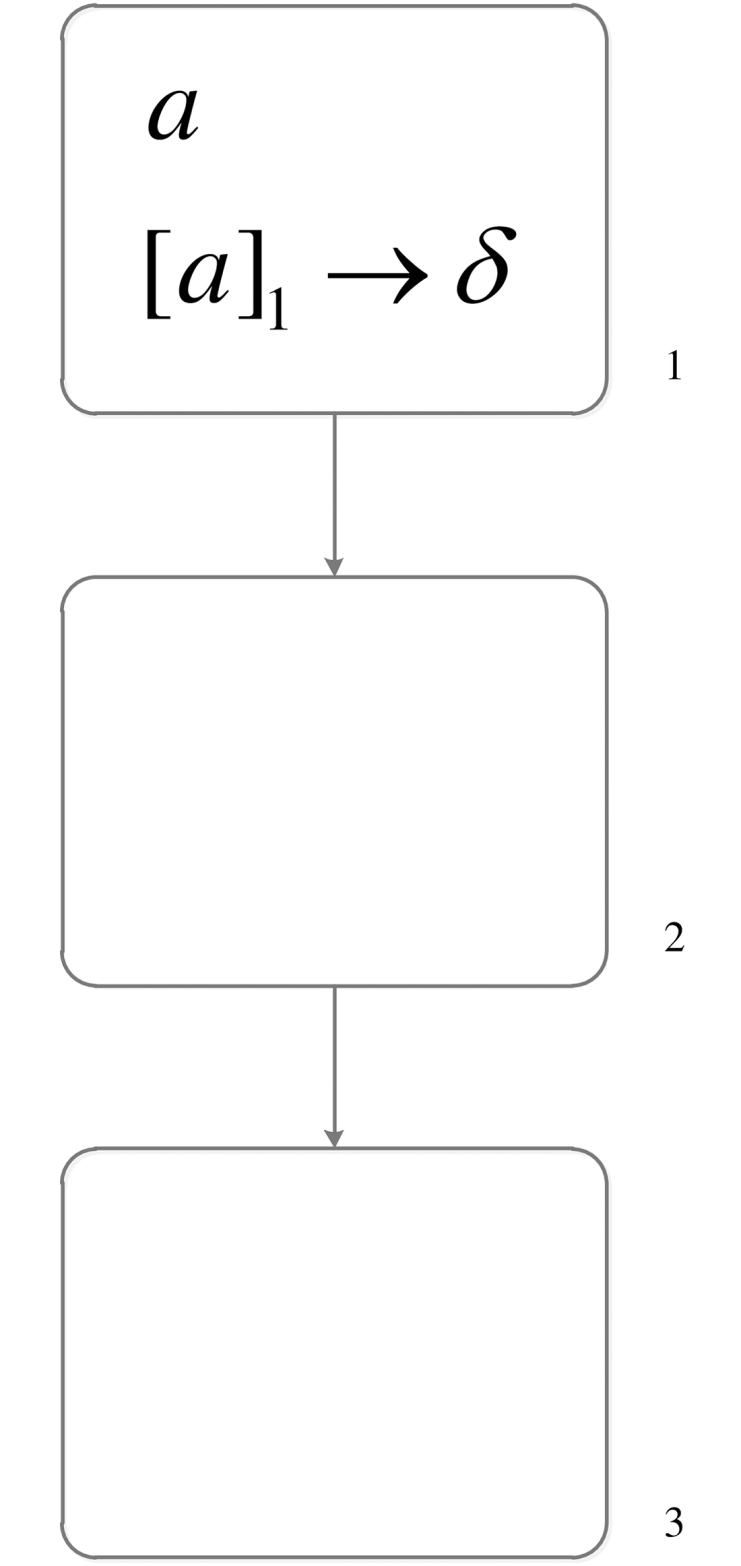
The SN P system before executing dissolution rule.

**Fig 4:**
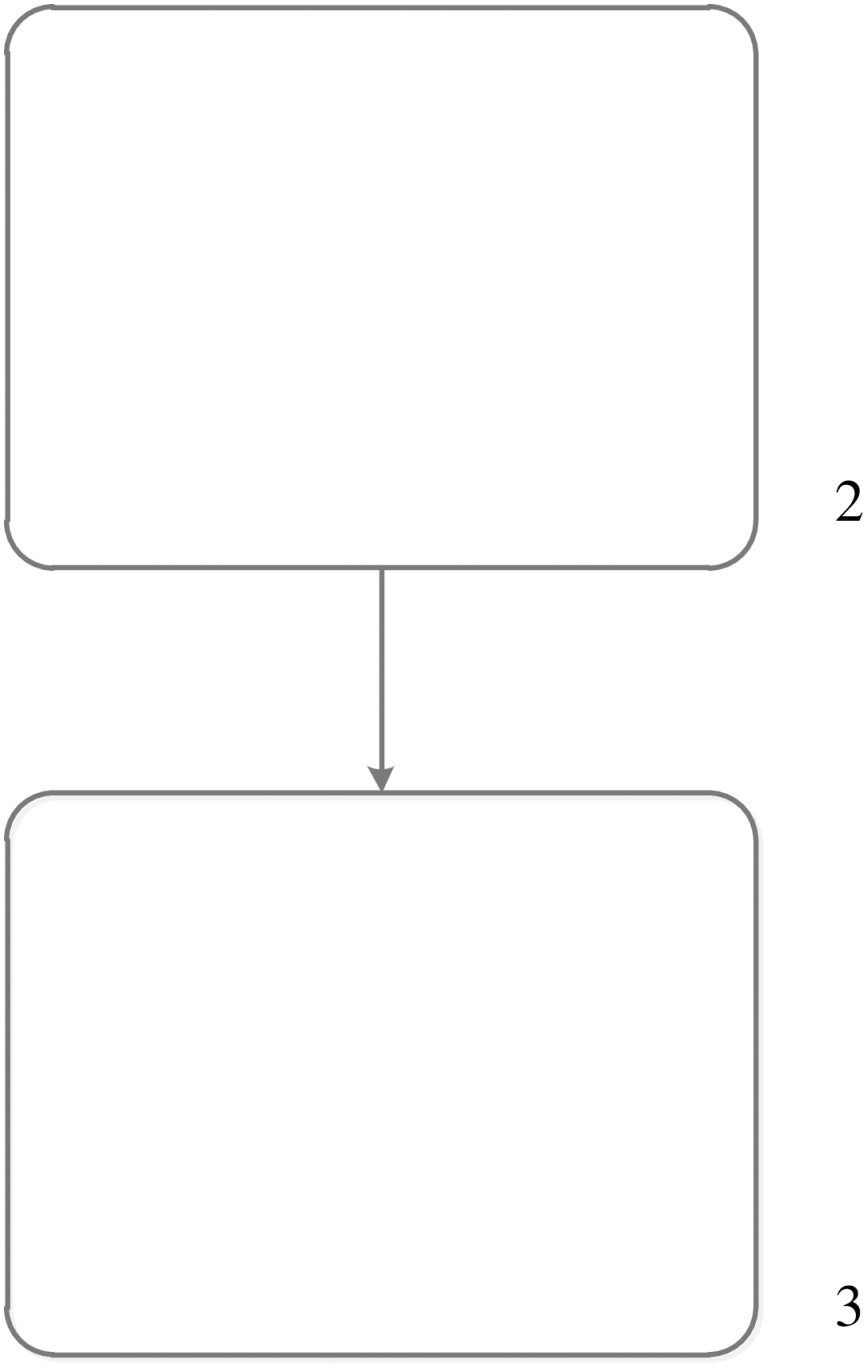
The SN P system after executing dissolution rule.

A simple example shown in Fig 3 is used to show how dissolution rules are applied. Neuron *σ*_1_ has one spike *a* and the regular expression of the dissolution rule is exactly {*a*}, where *a* ∈ {*a*}. Then rule [*a*]_1_ → *δ* is applied and the system is changed to Fig 4 (Neuron *σ*_1_ is dissolved, and synapse (1, 2) connected with neuron *σ*_1_ is also dissolved.).

At each step, if only one rule in neuron *σ*_*i*_ can be applied, this rule must be applied; if two or more rules in neuron *σ*_*i*_ can be applied, one of these rules is applied non-deterministically. Rules are applied in a sequential manner in each neuron and in parallel between neurons.

The *configuration* of the system is described by the synapses connections, the spikes number in each neuron, and the state of each neuron (open or closed). By applying rules, the configuration is *transformed* from one to the next one. The *transition* sequence starting from the initial configuration is called a *computation*, and a computation *halts* if it reaches a configuration where all neurons are open and no rule can be applied.

SN P systems can used to solve the decision problem *I*_*X*_, Θ_*X*_ both in a semi-uniform way and in a uniform way, where *I*_*X*_ is a language over a finite alphabet and Θ_*X*_ is a total boolean function over *I*_*X*_ (The elements in *I*_*X*_ are instances.). In the semi-uniform way, a specified SN P system is constructed for each instance of a decision problem, in which the instance parameters are embedded in the SN P system. In the uniform way, a SN P system is constructed for all instances of a decision problem, in which the different instances parameters enter the SN P system as input spikes. The uniform solutions are preferred because they only relate to the structure of a problem.

The input of a SN P system is a spike train $a^{i_{1}}\cdot a^{i_{2}}\cdot ...\cdot a^{i_{r}}$, where *r* ≥ 1, *i*_*j*_ ≥ 0 for each 1 ≤ *j* ≤ *r*, which means *i*_*j*_ spikes enter the system through input neuron *σ*_*in*_ at step *j*. Specially, *i*_*j*_ = 0 means no spike enters the system at step *j*.

## 2 A Uniform Solution to SAT Problem

SAT (the satisfiability of conjunctive normal form expression) problem is one of the most typical NP-complete problems. For a Boolean variable set *X* = {*x*_1_, *x*_2_, …, *x*_*n*_}, a literal *l*_*i*_ is *x*_*i*_ or ¬*x*_*i*_ for 1 ≤ *i* ≤ *n*. A clause *C*_*i*_ is a disjunction of literals *C*_*i*_ = *l*_*n*_1__ ∨ *l*_*n*_2__ ∨ … ∨ *l*_*n*_*r*__, 1 ≤ *r* ≤ *n*. A conjunctive normal form (CNF, for short) is a conjunction of clauses *C*_1_ ∧ *C*_2_ ∧ … ∧ *C*_*m*_. An assignment is a mapping *X* → {0, 1} from each variable *x*_*i*_ to its value (Value 1 represents true and value 0 represents false.). For example, *X* = {*x*_1_, *x*_2_, *x*_3_}, the conjunctive normal form is (*x*_1_ ∨ ¬*x*_2_) ∧ (*x*_1_ ∨ *x*_3_). The *x*_1_ ∨ ¬*x*_2_ and *x*_1_ ∨ *x*_3_ are the two clauses. The first clause contains two literals *x*_1_ and ¬*x*_2_, and the second clause contains two literals *x*_1_ and *x*_3_. If an assignment of *x*_1_, *x*_2_, …, *x*_*n*_ can be found, which makes at least one literal true in each clause and then makes all *m* clauses true, this SAT problem is satisfiable. Otherwise, this SAT problem is unsatisfiable [38]. In the above example, let *x*_1_ = *x*_2_ = *x*_3_ = 1, the value of the conjunctive normal form is (1 ∨ 0) ∧ (1 ∨ 0) = 1 ∧ 1 = 1. Therefore, the SAT problem is satisfiable.

The formal definition of SAT problem is as follows.

Problem 1. NAME: SAT.

- INSTANCE: a set of clauses *C* = {*C*_1_, *C*_2_, …, *C*_*m*_}, which is built on a Boolean variable set *X* = {*x*_1_, *x*_2_, …, *x*_*n*_}.
- QUESTION: is there an assignment of Boolean variables *x*_1_, *x*_2_, …, *x*_*n*_ that can make the value of all clauses true?

*SAT*(*n*, *m*) denotes the set of all instances of the SAT problem having *n* variables and *m* clauses. In this section, a uniform solution working in a deterministic way is constructed by DDSN P system, which can solve all *SAT*(*n*, *m*) problems in linear time.

The instance parameters need to enter a SN P system, therefore the clauses need to be encoded as spikes form. Each clause contains either *x*_*j*_, or ¬*x*_*j*_, or none of these two. Different numbers of spikes are introduced into the system to distinguish these three situations.

$$\alpha_{i,j}=\left\{\begin{array}{c} a,\;\;\text{if }x_{j}\text{ occurs in }C_{i};\\ a^{2},\;\text{if }\neg x_{j}\text{ occurs in }C_{i};\\ a^{0},\;\text{otherwise}. \end{array}\right.$$

A clause is represented by *α*_*i*,1_ ⋅ *α*_*i*,2_ ⋅ … ⋅ *α*_*i*,*n*_ in this way. For instance, a clause ¬*x*_1_⋁*x*_2_⋁*x*_3_ is represented by *a*^2^ ⋅ *a* ⋅ *a*.

In order to generate the necessary workspace before computing, a spike train (*a*^0^ ⋅)^2*n*^ is introduced into the front of each spike train.

**Fig 5:**
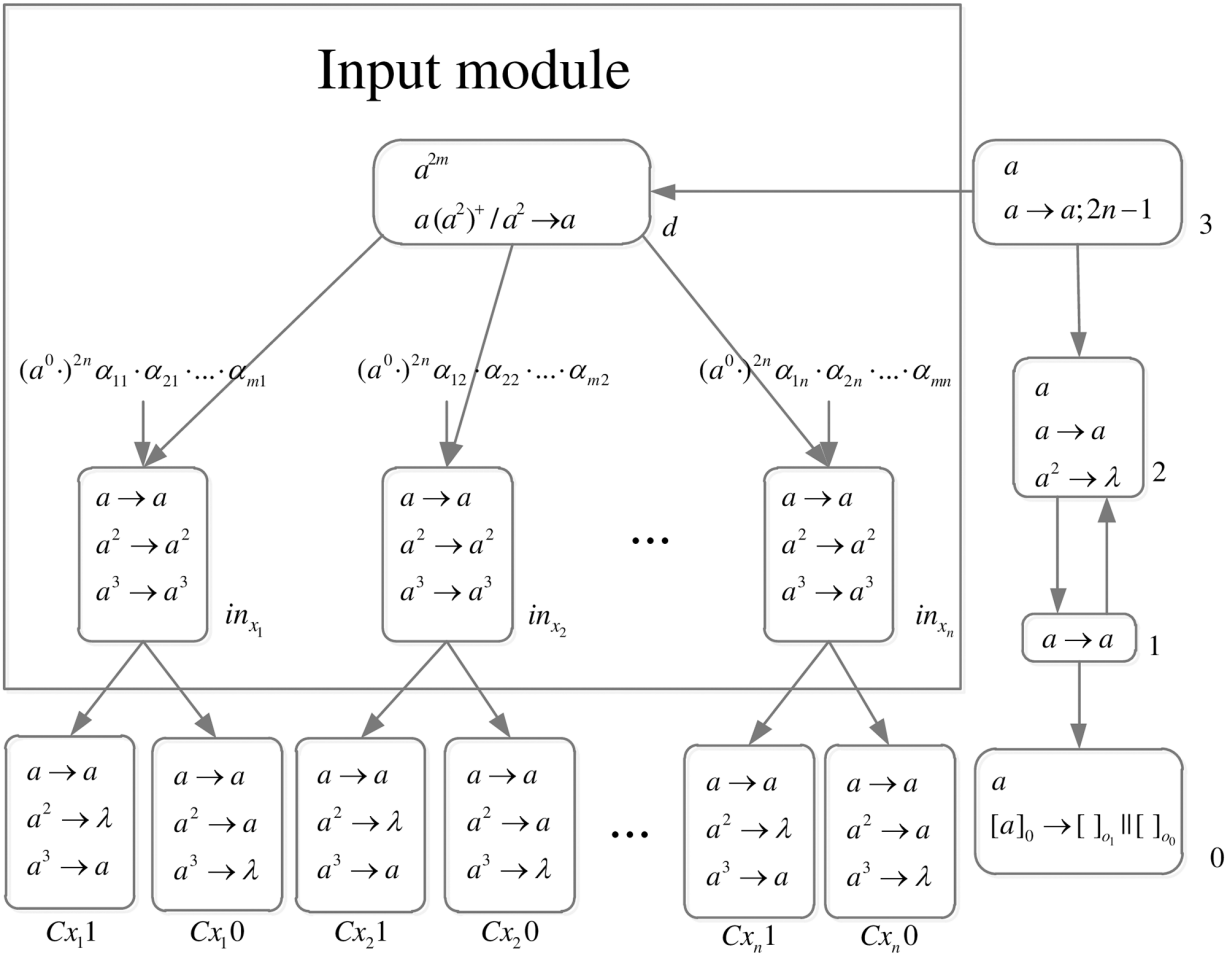
The initial system Π_*n*,*m*_.

The formal definition of DDSN P systems for *SAT*(*n*, *m*) problems (shown in Fig 5) is as follows.

$$\Pi_{n,m}=\left(O,H,syn,n_{1},n_{2},\ldots ,n_{3n+5},R,in,out\right),$$

where:

1. *O* = {*a*};
2. *H* = {0, 1, 2, 3, *d*, *in*_*x*_*i*__, *Cx*_*i*_1, *Cx*_*i*_0, *o*_*t*_1_, *t*_2_, …, *t*_*n*__}(*i*, *t*_1_, *t*_2_, …, *t*_*n*_ = 1,2);
3. *syn* = {(3, 2), (2, 1), (1, 2), (1, 0), (3, *d*)}
  ⋃{(*d*, *in*_*x*_*i*__)|*i* = 1, 2, …, *n*} ⋃{(*in*_*x*_*i*__, *Cx*_*i*_1), (*in*_*x*_*i*__, *Cx*_*i*_0)|*i* = 1, 2, …, *n*}
  ⋃{(*Cx*_1_1, *o*_1_), (*Cx*_*i*_1, *o*_*t*_1_, *t*_(*i* − 1)1__)|*i* = 2, 3, …, *n*}
  ⋃{(*Cx*_1_0, *o*_0_), (*Cx*_*i*_0, *o*_*t*_1_, *t*_(*i* − 1)0__)|*i* = 2, 3, …, *n*};
4. *n*_0_ = 1, *n*_2_ = 1, *n*_3_ = 1, *n*_*d*_ = 2*m*, the number of spikes in other neurons is zero;
5. *in* = *σ*_*in*_*x*_*i*___, *out* = *σ*_*o*_*t*_1_, *t*_2_, …, *t*_*n*___(*i*, *t*_1_, *t*_2_, …, *t*_*n*_ = 0, 1);
6. **firing rule:**
  [*a* → *a*]_*i*_, *i* = 1, 2
  [*a* → *a*; 2*n* − 1]_3_
  [*a*(*a*^2^)^+^/*a*^2^ → *a*]_*d*_
  [*a* → *a*]_*in*_*x*_*i*___, *i* = 1, 2, …, *n*
  [*a*^2^ → *a*^2^]_*in*_*x*_*i*___, *i* = 1, 2, …, *n*
  [*a*^3^ → *a*^3^]_*in*_*x*_*i*___, *i* = 1, 2, …, *n*
  [*a* → *a*]_*Cx*_*i*_1_, *i* = 1, 2, …, *n*
  [*a*^3^ → *a*]_*Cx*_*i*_1_, *i* = 1, 2, …, *n*
  [*a* → *a*]_*Cx*_*i*_0_, *i* = 1, 2, …, *n*
  [*a*^2^ → *a*]_*Cx*_*i*_0_, *i* = 1, 2, …, *n*
  **forgetting rule:**
  [*a*^2^ → λ]_2_
  [*a*^2^ → λ]_*Cx*_*i*_1_, *i* = 1, 2, …, *n*
  [*a*^3^ → λ]_*Cx*_*i*_0_, *i* = 1, 2, …, *n*
  [*a* → λ]_*o*_*t*_1_*t*_2_…*t*_*n*___, *t*_1_, *t*_2_, …, *t*_*n*_ = 0, 1
  [*a*^2^ → λ]_*o*_*t*_1_*t*_2_…*t*_*n*___, *t*_1_, *t*_2_, …, *t*_*n*_ = 0, 1
  …
  [*a*^*n* −1^ → λ]_*o*_*t*_1_*t*_2_…*t*_*n*___, *t*_1_, *t*_2_, …, *t*_*n*_ = 0, 1
  **neuron division rule:**
  [*a*]_0_ → [ ]_*o*_1__ || [ ]_*o*_0__
  [*a*]_*o*_*t*_1___ → [ ]_*o*_*t*_1_1__ || [ ]_*o*_*t*_1_0__, *t*_1_ = 0, 1
  [*a*]_*o*_*t*_1_*t*_2___ → [ ]_*o*_*t*_1_*t*_2_1__ || [ ]_*o*_*t*_1_*t*_2_0__, *t*_1_, *t*_2_ = 0, 1
  …
  [*a*]_*o*_*t*_1_*t*_2_…*t*_*n*−1___ → [ ]_*o*_*t*_1_*t*_2_…*t*_*n*−1_1__ || [ ]_*o*_*t*_1_*t*_2_…*t*_*n*−1_0__, *t*_1_, *t*_2_, …, *t*_*n*−1_ = 0, 1
  **neuron dissolution rule:**
  [*a*^*n*^]_*o*_*t*_1_*t*_2_…*t*_*n*___ → *δ*, *t*_1_, *t*_2_, …, *t*_*n*_ = 0, 1.

Computation starts when spike trains enter the system through input neurons *σ*_*in*_*x*_1___, *σ*_*in*_*x*_2___, …, *σ*_*in*_*x*_*n*___, respectively. Neuron *σ*_0_ and its children neurons need 2*n* steps to generate 2^*n*^ neurons (workspace) to enumerate all assignments of variables by applying neuron division rules (One neuron represents one assignment of variables.), therefore (*a*^0^ ⋅)^2*n*^ are added to the front of each spike train.

**Fig 6:**
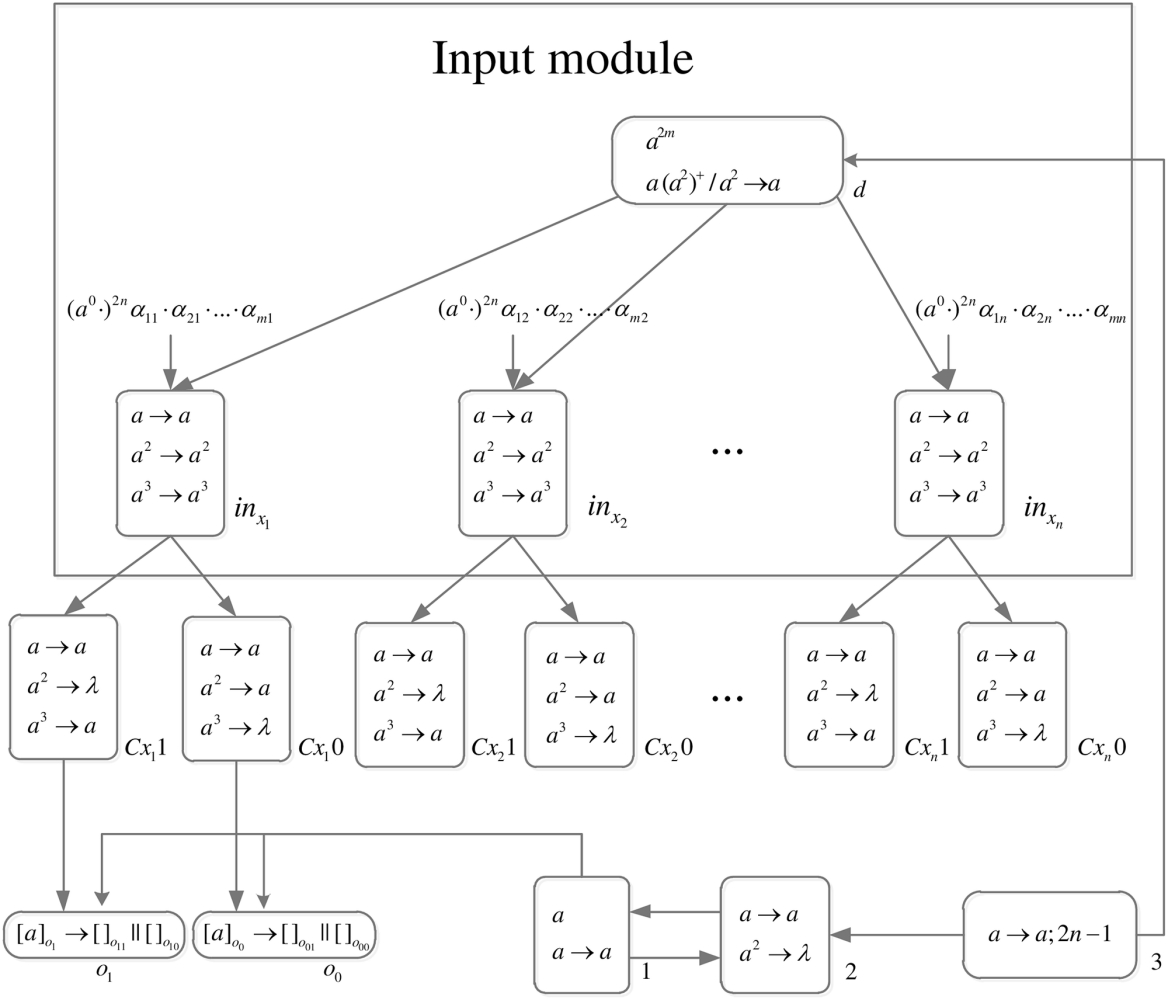
The system Π_*n*,*m*_ after step one.

*Generation Stage*: At step one, neuron *σ*_0_ has one spike, the division rule [*a*]_0_ → [ ]_*o*_1__ || [ ]_*o*_0__ is applied to generate neurons *σ*_*o*_1__ and *σ*_*o*_0__, which means an assignment in regard to *x*_1_ has two choices: 1 or 0. Synapses (1, *o*_1_) and (1, *o*_0_) are established through the inheritance of synapse (1, 0), and synapses (*Cx*_1_1, *o*_1_) and (*Cx*_1_0, *o*_0_) are established through synapse dictionary *syn*. Synapse (*Cx*_1_1, *o*_1_) establishes a channel between the input and the assignment including *x*_1_ = 1; synapse (*Cx*_1_0, *o*_0_) establishes a channel between the input and the assignment including *x*_1_ = 0. At the same time, auxiliary neuron *σ*_2_ has one spike, rule *a* → *a* is applied and one spike is emitted to neuron *σ*_1_; auxiliary neuron *σ*_3_ has one spike, rule *a* → *a*; 2*n* − 1 is applied and one spike will be emitted to neurons *σ*_2_ and *σ*_*d*_ at step 2*n*. The system after step one is shown in Fig 6.

At step two, neuron *σ*_1_ has one spike, the firing rule *a* → *a* is applied, and one spike is emitted to neurons *σ*_2_, *σ*_*o*_1__ and *σ*_*o*_0__.

**Fig 7:**
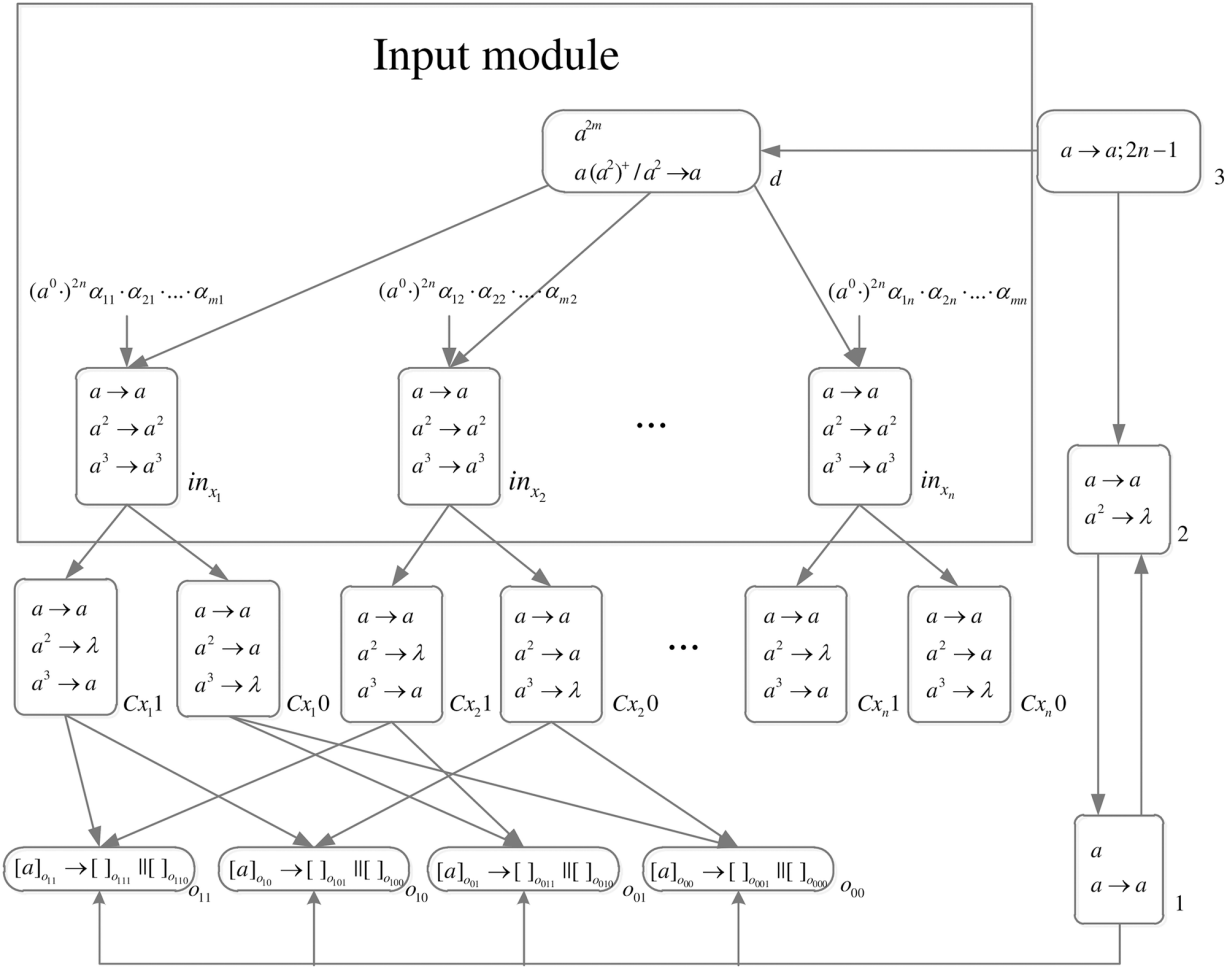
The system Π_*n*,*m*_ after step three.

At step three, each of neurons *σ*_*o*_1__ and *σ*_*o*_0__ has one spike, the division rule [*a*]_*o*_*t*_1___ → [ ]_*o*_*t*_1_1__ || [ ]_*o*_*t*_1_0__(*t*_1_ = 1, 0) is applied to generate neurons *σ*_*o*_11__, *σ*_*o*_10__, *σ*_*o*_01__ and *σ*_*o*_00__, which means an assignment in regard to *x*_1_ and *x*_2_ has four choices: 11, 10, 01, 00. Synapses (1, *o*_11_), (1, *o*_10_), (1, *o*_01_) and (1, *o*_00_) are established through the inheritance of synapses (1, *o*_1_), (1, *o*_0_); synapses (*Cx*_1_1, *o*_11_), (*Cx*_1_1, *o*_10_), (*Cx*_1_0, *o*_01_) and (*Cx*_1_1, *o*_00_) are established through the inheritance of synapses (*Cx*_1_1, *o*_1_), (*Cx*_1_0, *o*_0_); synapses (*Cx*_2_1, *o*_11_), (*Cx*_2_1, *o*_01_), *S*(*Cx*_2_0, *o*_10_) and (*Cx*_2_0, *o*_00_) are established through synapse dictionary *syn*. Synapses (*Cx*_1_1, *o*_11_) and (*Cx*_1_1, *o*_10_) establish channels between the input and the assignments including *x*_1_ = 1; synapses (*Cx*_1_0, *o*_01_) and (*Cx*_1_0, *o*_00_) establish channels between the input and the assignments including *x*_1_ = 0; synapses (*Cx*_2_1, *o*_11_) and (*Cx*_2_1, *o*_01_) establish channels between the input and the assignments including *x*_2_ = 1; synapses (*Cx*_2_0, *o*_10_) and (*Cx*_2_0, *o*_00_) establish channels between the input and the assignments including *x*_2_ = 0. At the same time, auxiliary neuron *σ*_2_ has one spike, rule *a* → *a* is applied and one spike is emitted to neuron *σ*_1_. The system after step three is shown in Fig 7.

**Fig 8:**
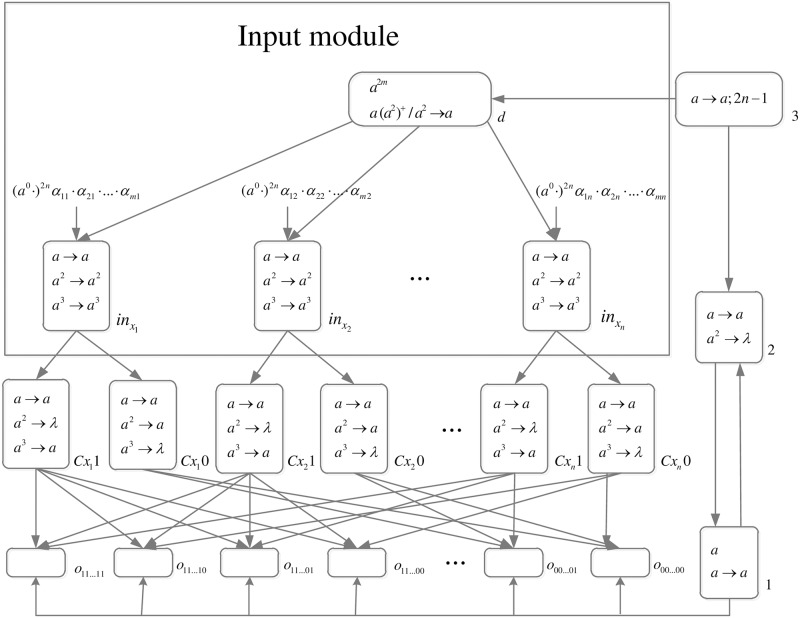
The system Π_*n*,*m*_ after step 2*n* − 1.

Similar process repeats. At step 2*n* − 1, 2^*n*^ neurons labeled *o*_*t*_1_*t*_2_…*t*_*n*__(*t*_1_, *t*_2_, …, *t*_*n*_ = 0, 1) are generated. The system after step 2*n* − 1 is shown in Fig 8.

**Fig 9:**
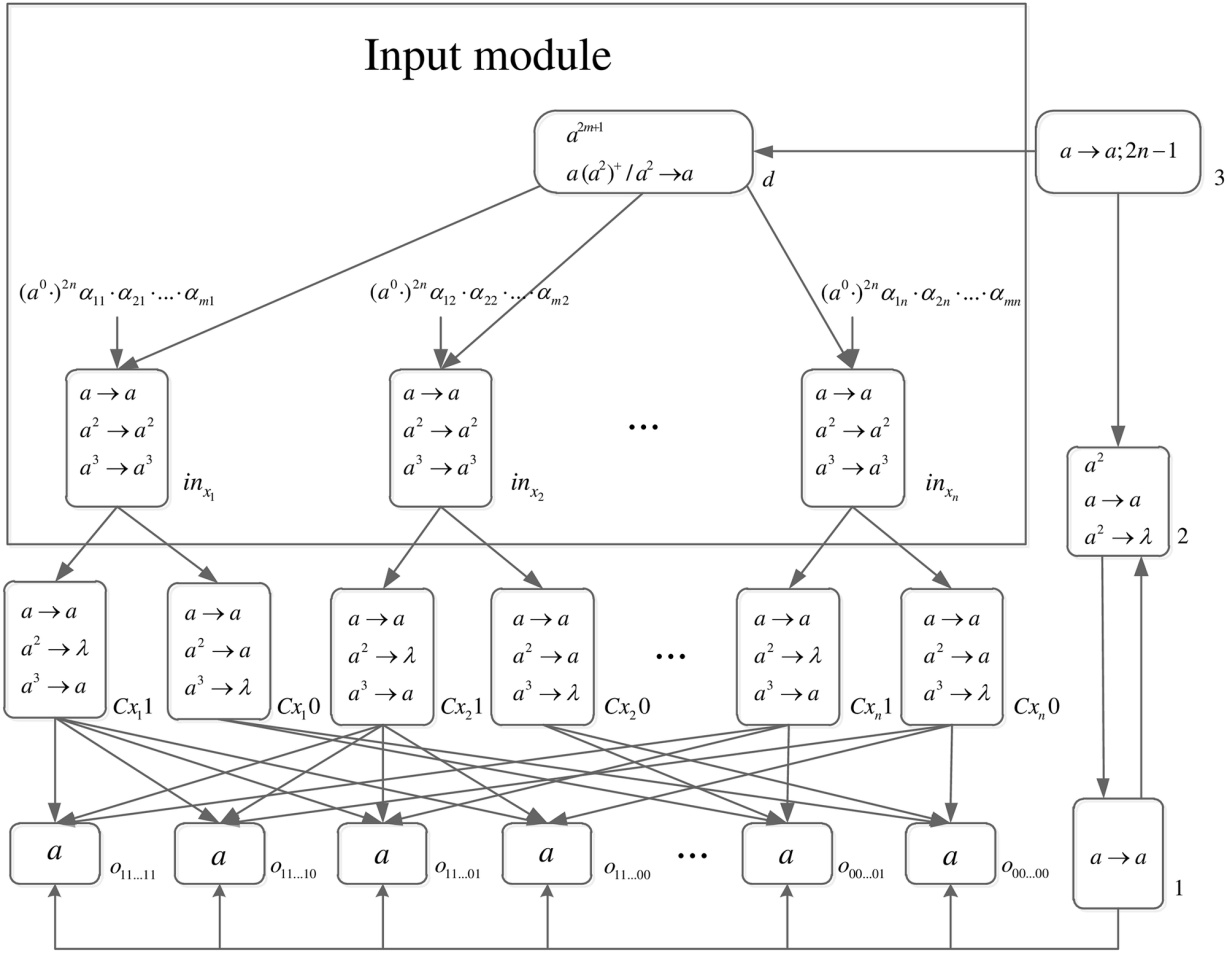
The system Π_*n*,*m*_ after step 2*n*.

At step 2*n*, each neuron *σ*_*o*_*t*_1_*t*_2_…*t*_*n*___ receives one spike emitted from neuron *σ*_1_ which will be deleted at the next step by the forgetting rule [*a* → λ]_*o*_*t*_1_*t*_2_…*t*_*n*___. Neuron *σ*_2_ receives two spikes (One is emitted from neuron *σ*_1_, and another one is emitted from *σ*_3_), the forgetting rule *a*^2^ → λ is applied at step 2*n* + 1, and no spike will be emitted to neuron *σ*_1_ later. At the same time, neuron *σ*_*d*_ receives one spike emitted from *σ*_3_. The system after step 2*n* is shown in Fig 9.

*Input Stage*: At step 2*n* + 1, the first clause of the conjunctive normal form expression enters the system through input neurons *σ*_*in*_*x*_*i*___, *i* = 1, 2, …, *n*. The literal in regard to *x*_1_ enters neuron *σ*_*in*_*x*_1___; the literal in regard to *x*_2_ enters neuron *σ*_*in*_*x*_2___; … the literal in regard to *x*_*n*_ enters neuron *σ*_*in*_*x*_*n*___. At the same time, one spike is emitted to neuron *σ*_*in*_*x*_*i*___ from neuron *σ*_*d*_.

At step 2*n* + 2, the spikes in neuron *σ*_*in*_*x*_*i*___ are replicated, and are emitted to neurons *σ*_*Cx*_*i*_1_ and *σ*_*Cx*_*i*_0_.

At step 2*n* + 3, different rules are applied according to the number of spikes in neurons *σ*_*Cx*_*i*_1_ and *σ*_*Cx*_*i*_0_.

For neuron *σ*_*Cx*_*i*_1_:

- If one spike is in neuron *σ*_*Cx*_*i*_1_, which means neither *x*_*i*_ nor ¬*x*_*i*_ is in the clause, rule *a* → *a* is applied. One spike is emitted to neurons having synapses going from neuron *σ*_*Cx*_*i*_1_ to them. It aims to show that *x*_*i*_ = 1 makes no contribution to let the clause true.
- If two spikes are in neuron *σ*_*Cx*_*i*_1_, which means *x*_*i*_ is in the clause, rule *a*^2^ → λ is applied. These two spikes are deleted. It aims to show that *x*_*i*_ = 1 makes contribution to let the clause true.
- If three spikes are in neuron *σ*_*Cx*_*i*_1_, which means ¬*x*_*i*_ is in the clause, rule *a*^3^ → *a* is applied. One spike is emitted to neurons having synapses going from neuron *σ*_*Cx*_*i*_1_ to them. It aims to show that *x*_*i*_ = 1 makes no contribution to let the clause true.

For neuron *σ*_*Cx*_*i*_0_:

- If one spike is in neuron *σ*_*Cx*_*i*_0_, which means neither *x*_*i*_ nor ¬*x*_*i*_ is in the clause, rule *a* → *a* is applied. One spike is emitted to neurons having synapses going from neuron *σ*_*Cx*_*i*_0_ to them. It aims to show that *x*_*i*_ = 0 makes no contribution to let the clause true.
- If two spikes are in neuron *σ*_*Cx*_*i*_0_, which means *x*_*i*_ is in the clause, rule *a*^2^ → *a* is applied. One spike is emitted to neurons having synapses going from neuron *σ*_*Cx*_*i*_0_ to them. It aims to show that *x*_*i*_ = 0 makes no contribution to let the clause true.
- If three spikes are in neuron *σ*_*Cx*_*i*_0_, which means ¬*x*_*i*_ is in the clause, rule *a*^3^ → λ is applied. These three spikes are deleted. It aims to show that *x*_*i*_ = 0 makes contribution to let the clause true.

*Satisfiability Stage*: Each neuron *σ*_*o*_*t*_1_*t*_2_…*t*_*n*___(*t*_1_, *t*_2_, …, *t*_*n*_ = 0, 1) receives zero or more spikes at step 2*n* + 3. If one neuron *σ*_*o*_*t*_1_*t*_2_…*t*_*n*___ receives *n* spikes which means the clause contains *n* literals that make no contribution to let the clause true, the dissolution rule [*a*^*n*^]_*o*_*t*_1_*t*_2_…*t*_*n*___ → *δ* is applied at step 2*n* + 4 to dissolve this neuron (The value of the first clause is false, therefore this assignment is not the answer to this SAT problem.). Otherwise, at least one literal is true in this assignment and this assignment is reserved to check the next clause.

Due to *m* clauses are in a SAT problem, the satisfiability checking stage lasts for *m* + 3 steps. If some neurons *σ*_*o*_*t*_1_*t*_2_…*t*_*n*___ are still in the system at step 2*n* + *m* + 3, the labels of these neurons *σ*_*o*_*t*_1_*t*_2_…*t*_*n*___ are all solutions to this SAT problem, i.e., this SAT problem is satisfiable. Otherwise, this SAT problem is unsatisfiable.

It can be seen that any *SAT*(*n*, *m*) problem can be solved in linear time, and all solutions can be obtained through this system.

Some steps comparison results between our solution and other solutions, which use the neuron division to solve the NP-complete problems, are shown in Table 1.

**Table 1:** Steps comparison results of some uniform solutions to SAT problem.

solution	step complexity (steps)	determinism or nondeterminism
SN P systems with neuron division and budding [25]	2*n* + *mn* + 6	determinism
SN P systems with neuron division [26]	4*n* + *mn* + 5	determinism
**DDSN P systems**	2*n* + *m* + 3	determinism

Considering a SAT problem *SAT (3, 3)*: (*x*_1_ ⋁ *x*_2_) ⋀ (¬*x*_2_ ⋁ *x*_3_) ⋀ (¬*x*_1_ ⋁ *x*_2_ ⋁ *x*_3_), the DDSN P system Π_3,3_ is used to solve it. After 12 computational steps, neurons *σ*_*o*_111__, *σ*_*o*_101__ and *σ*_*o*_011__ are remaining which shows that {*x*_1_ = *true*, *x*_2_ = *true*, *x*_3_ = *true*}, {*x*_1_ = *true*, *x*_2_ = *false*, *x*_3_ = *true*} and {*x*_1_ = *false*, *x*_2_ = *true*, *x*_3_ = *true*} are all solutions to this SAT problem.

The SN P system with neuron division and budding and the SN P system with neuron division need 21 steps and 26 steps to judge this problem has solutions, respectively, while our DDSN P system need only 12 steps.

**Fig 10:**
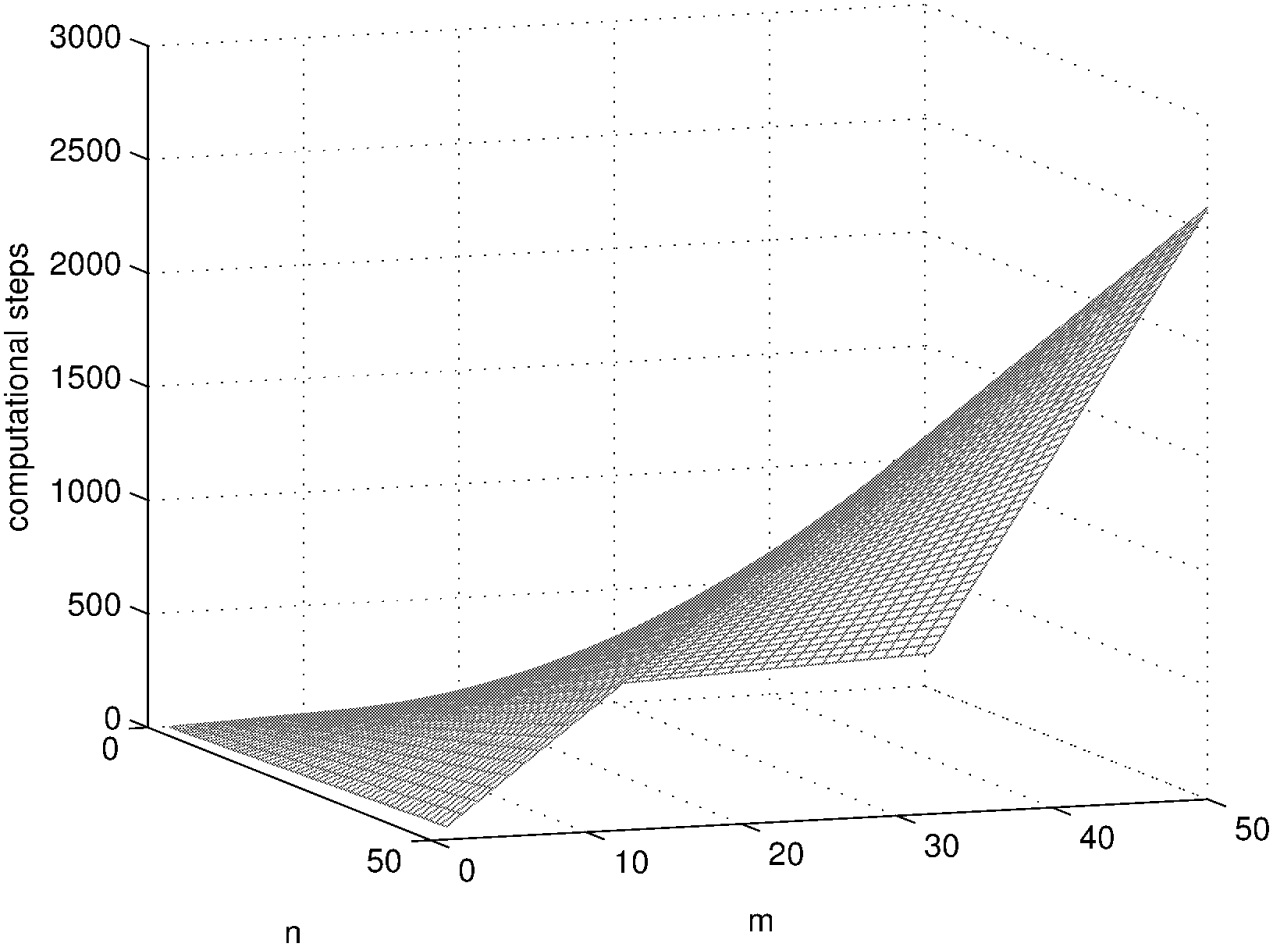
The computational steps of SN P system with neuron division and budding solving SAT problem.

**Fig 11:**
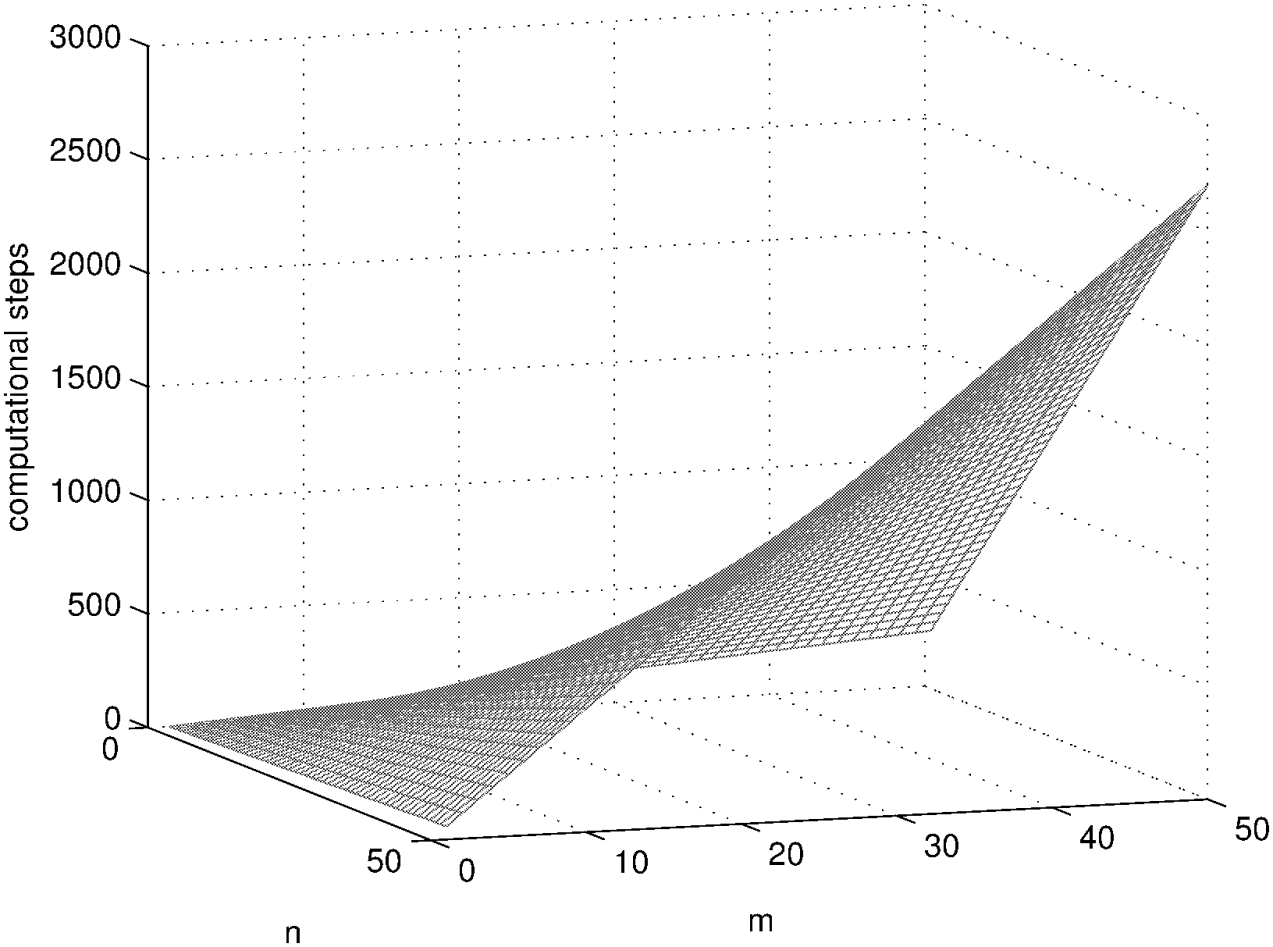
The computational steps of SN P system with neuron division solving SAT problem.

**Fig 12:**
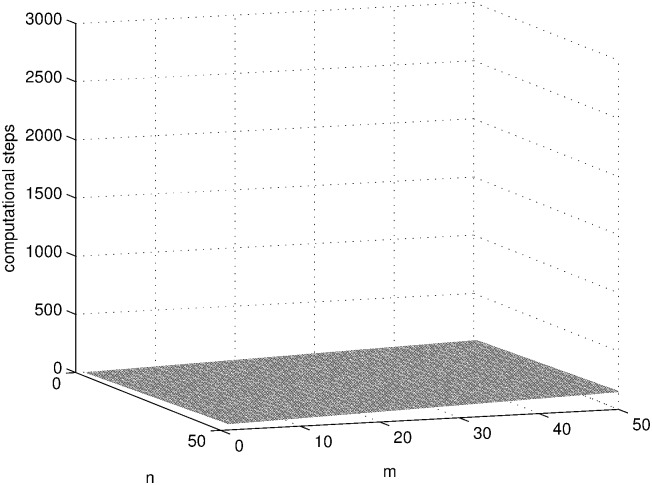
The computational steps of DDSN P system solving SAT problem.

SAT problems with different sizes (1 ≤ *n*, *m* ≤ 50) are solved using the three systems in Table 1 and the computational steps of each system are shown in Figs 10, 11 and 12 by MATLAB R2014a. As can be seen from these figures, the computational steps of DDSN P system are stable and much fewer, especially when the problem size is larger.

## 3 A Uniform Solution to Subset Sum Problem

Subset Sum problem is one of the most typical NP-complete problems. The formal definition of it is as follows [38].

Problem 2. NAME: SUBSET SUM.

- INSTANCE: a set of positive integers *X* = {*x*_1_, *x*_2_, …, *x*_*n*_} and a positive integer *S*.
- QUESTION: is there a subset *B* ⊆ *X* that $\sum_{b\in B}b=S$?

*Subset* *Sum* *problem* (*n*) denotes the set of all instances of the Subset Sum problem having *n* integers. In this section, a uniform solution working in a deterministic way is constructed by DDSN P system, which can solve all *Subset* *Sum* *problem* (*n*) problems in linear time.

An integer is represented by corresponding number of spikes. In order to generate necessary workspace before computing, a spike train (*a*^0^ ⋅)^2*n*^ is introduced into the front of each spike train.

**Fig 13:**
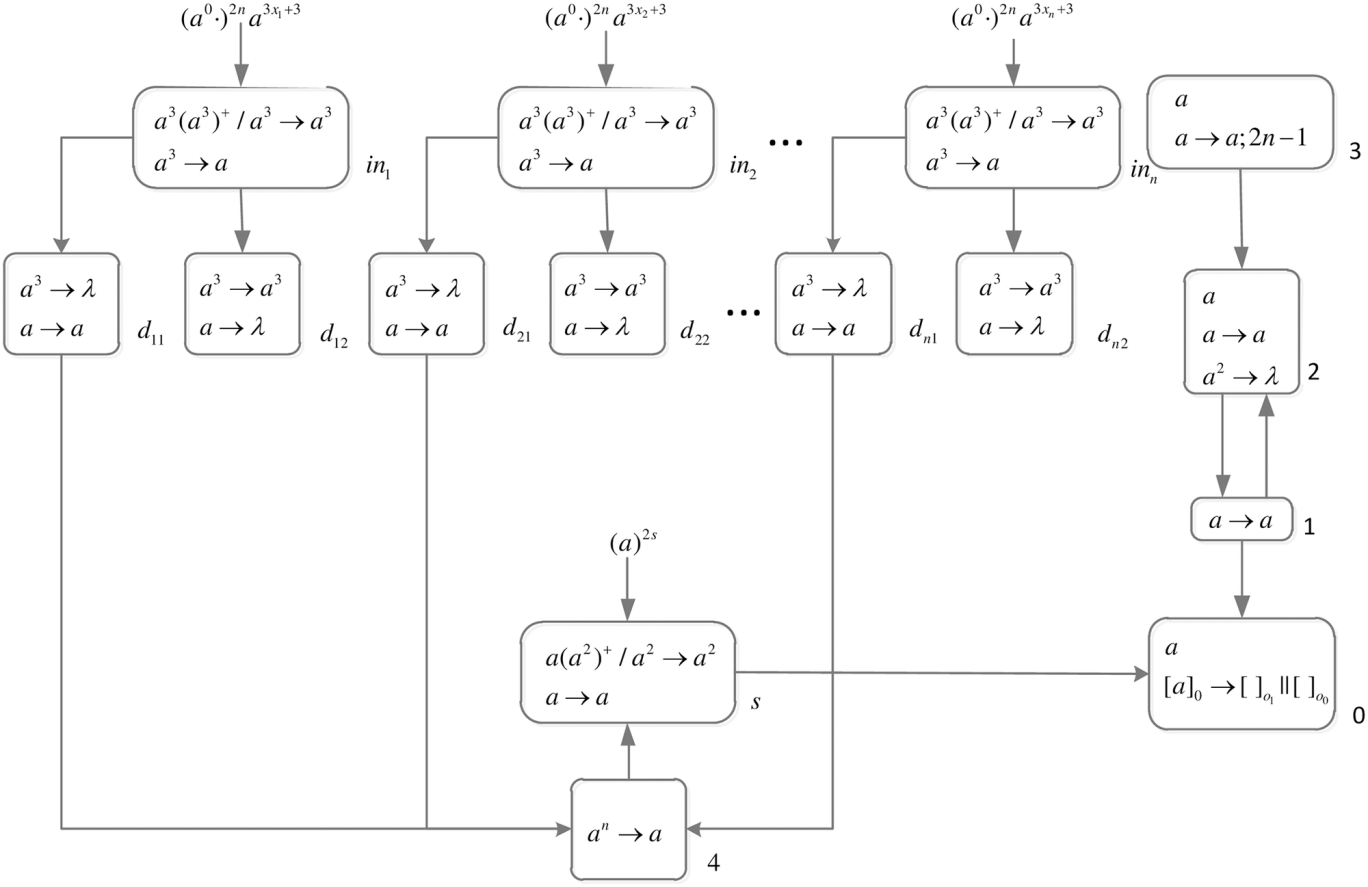
The initial system Π_*n*_.

The formal definition of DDSN P Systems for *Subset* *Sum* *problem* (*n*) (shown in Fig 13) is as follows.

$$\Pi_{n}=\left(O,H,syn,n_{1},n_{2},\ldots ,n_{3n+6},R,in,out\right),$$

where:

1. *O* = {*a*};
2. *H* = {0, 1, 2, 3, 4, *s*, *in*_*i*_, *d*_*i*1_, *d*_*i*2_}(*i* = 1, 2, …, *n*);
3. *syn* = {(3, 2), (2, 1), (1, 2), (1, 0), (4, *s*), (*s*, 0)}
  ⋃{(*in*_*i*_, *d*_*i*1_), (*in*_*i*_, *d*_*i*2_), (*d*_*i*1_, 4)|*i* = 1, 2, …, *n*}
  ⋃{(*d*_1,2_, *o*_1_)}⋃{(*d*_*i*2_, *o*_*t*_1_, …, *t*_(*i* − 1)_1_)|*i* = 2, 3, …, *n*};
4. *n*_0_ = 1, *n*_2_ = 1, *n*_3_ = 1, the number of spikes in other neurons is zero;
5. *in* = *σ*_*in*_*i*__, *σ*_*s*_, *out* = *σ*_*o*_*t*_1_, *t*_2_, …, *t*_*n*___(*i*, *t*_1_, *t*_2_, …, *t*_*n*_ = 0, 1);
6. **firing rule:**
  [*a* → *a*]_*i*_, *i* = 1, 2
  [*a* → *a*; 2*n* − 1]_3_
  [*a*^3^(*a*^3^)^+^/*a*^3^ → *a*^3^]_*in*_*i*__, *i* = 1, 2, …, *n*
  [*a*^3^ → *a*]_*in*_*i*__, *i* = 1, 2, …, *n*
  [*a* → *a*]_*d*_*i*1__, *i* = 1, 2, …, *n*
  [*a*^3^ → *a*^3^]_*d*_*i*2__, *i* = 1, 2, …, *n*
  [*a*^*n*^ → *a*]_4_
  [*a*(*a*^2^)^+^/*a*^2^ → *a*^2^]_*s*_
  [*a* → *a*]_*s*_
  **forgetting rule:**
  [*a*^2^ → λ]_2_
  [*a*^3^ → λ]_*d*_*i*1__, *i* = 1, 2, …, *n*
  [*a* → λ]_*d*_*i*2__, *i* = 1, 2, …, *n*
  [*a*^2^(*a*^3^)^+^/*a*^5^ → λ]_*o*_*t*_1_*t*_2_…*t*_*n*___, *t*_1_, *t*_2_, …, *t*_*n*_ = 0, 1
  [*a* → λ]_*o*_*t*_1_*t*_2_…*t*_*n*___, *t*_1_, *t*_2_, …, *t*_*n*_ = 0, 1
  **neuron division rule:**
  [*a*]_0_ → [ ]_*o*_1__ || [ ]_*o*_0__
  [*a*]_*o*_*t*_1___ → [ ]_*o*_*t*_1_1__ || [ ]_*o*_*t*_1_0__, *t*_1_ = 0, 1
  [*a*]_*o*_*t*_1_*t*_2___ → [ ]_*o*_*t*_1_*t*_2_1__ || [ ]_*o*_*t*_1_*t*_2_0__, *t*_1_, *t*_2_ = 0, 1
  …
  [*a*]_*o*_*t*_1_*t*_2_…*t*_*n*−1___ → [ ]_*o*_*t*_1_*t*_2_…*t*_*n*−1_1__ || [ ]_*o*_*t*_1_*t*_2_…*t*_*n*−1_0__, *t*_1_, *t*_2_, …, *t*_*n*−1_ = 0, 1
  **neuron dissolution rule:**
  [*a*(*a*^3^)^+^]_*o*_*t*_1_*t*_2_…*t*_*n*___ → *δ*, *t*_1_, *t*_2_, …, *t*_*n*_ = 0, 1
  [*a*^2^]_*o*_*t*_1_*t*_2_…*t*_*n*___ → *δ*, *t*_1_, *t*_2_, …, *t*_*n*_ = 0, 1.

Computation starts when spike trains enter the system thrgouth input neurons *σ*_*in*_1__, *σ*_*in*_2__, …, *σ*_*in*_*n*__ and *σ*_*s*_, respectively. Neuron *σ*_0_ and its children neurons need 2*n* steps to generate 2^n^ neurons (workspace) to enumerate all subsets of *x*_1_, *x*_2_, …, *x*_*n*_ by applying neuron division rules (One neuron represents one subset.), therefore (*a*^0^ ⋅)^2*n*^ are added to the front of each spike train.

**Fig 14:**
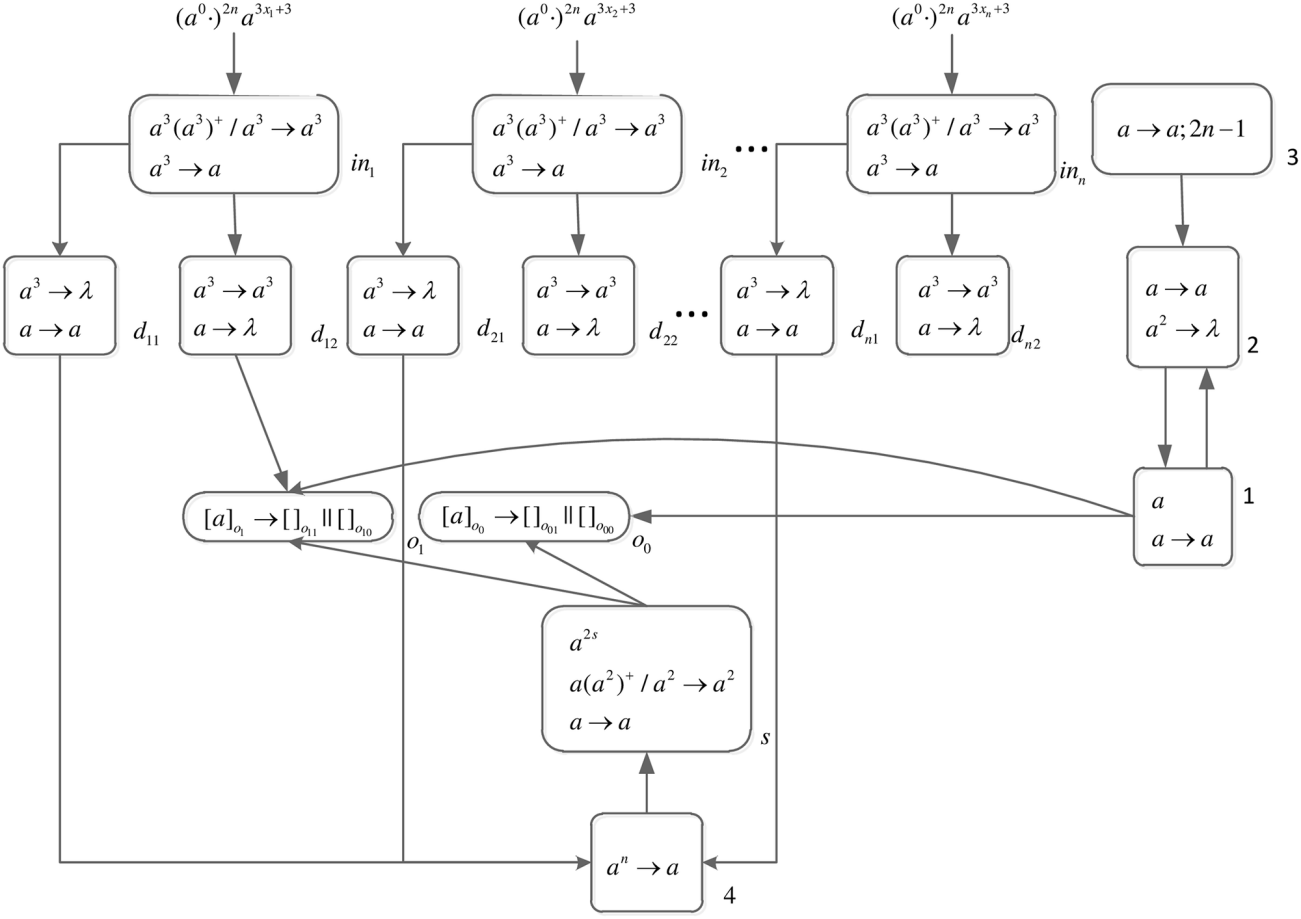
The system Π_*n*_ after step one.

*Generation Stage*: At step one, neuron *σ*_0_ has one spike, the division rule [*a*]_0_ → [ ]_*o*_1__ || [ ]_*o*_0__ is applied to generate neurons *σ*_*o*_1__ and *σ*_*o*_0__, which means one subset in regard to *x*_1_ has two choices: *x*_1_ is included in this subset (represent by 1) and *x*_1_ is not included in this subset (represent by 0). Synapses (1, *o*_1_) and (1, *o*_0_) are established through the inheritance of synapse (1, 0); synapses (*s*, *o*_1_) and (*s*, *o*_0_) are established through the inheritance of synapse (*s*, 0); the synapse (*d*_12_, *o*_1_) is established through synapse dictionary *syn*. The synapse between neurons *d*_12_ and *o*_1_ establishes a channel between the input and the subset having *x*_1_. At the same time, auxiliary neuron *σ*_2_ has one spike, rule *a* → *a* is applied and one spike is emitted to neuron *σ*_1_; auxiliary neuron *σ*_3_ has one spike, rule*a* → *a*; 2*n* − 1 is applied and one spike will be emitted to neurons *σ*_2_ at step 2*n*. The system after step one is shown in Fig 14.

At step two, neuron *σ*_1_ has one spike, the firing rule *a* → *a* is applied, and one spike is emitted to neurons *σ*_2_, *σ*_*o*_1__ and *σ*_*o*_0__.

**Fig 15:**
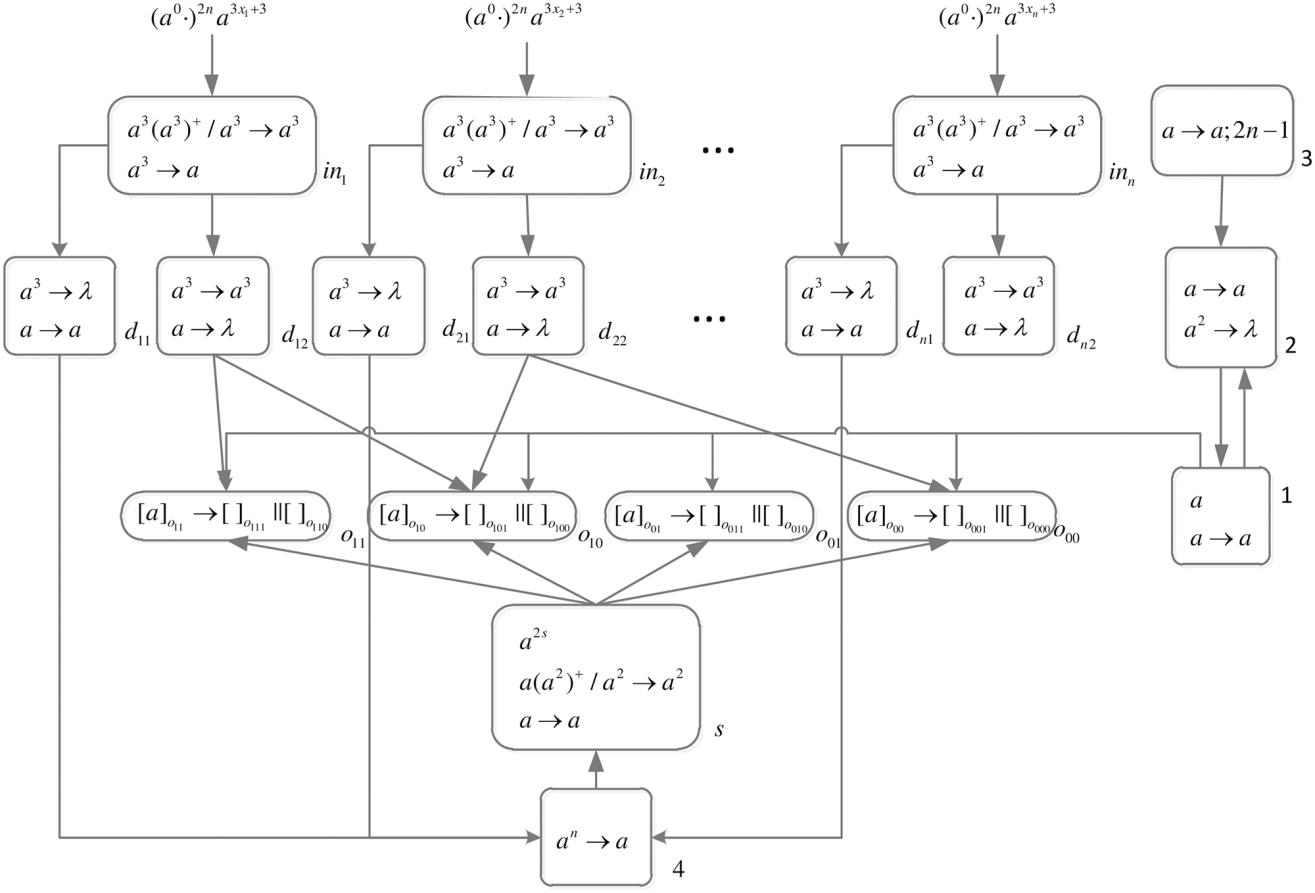
The system Π_*n*_ after step three.

At step three, each of neurons *σ*_*o*_1__ and *σ*_*o*_0__ has one spike, the division rule [*a*]_*o*_*t*_1___ → [ ]_*o*_*t*_1_1__ || [ ]_*o*_*t*_1_0__(*t*_1_ = 1, 0) is applied to generate neurons *σ*_*o*_11__, *σ*_*o*_10__, *σ*_*o*_01__ and *σ*_*o*_00__, which means one subset in regard to *x*_1_ and *x*_2_ has four choices: *x*_1_*x*_2_ are included in this subset (represent by 11), *x*_1_ is included in this subset and *x*_2_ is not included in this subset (represent by 10), *x*_1_ is not included in this subset and *x*_2_ is included in this subset (represent by 01), and *x*_1_*x*_2_ are not included in this subset (represent by 00). Synapses (1, *o*_11_), (1, *o*_10_), (1, *o*_01_), (1, *o*_00_), (*s*, *o*_11_), (*s*, *o*_10_), (*s*, *o*_01_), (*s*, *o*_00_), (*d*_12_, *o*_11_) and (*d*_12_, *o*_10_) are established through the inheritance of synapse (1, *o*_1_), (1, *o*_0_), (*s*, *o*_1_), (*s*, *o*_0_) and (*d*_12_, *o*_1_); synapses (*d*_22_, *o*_11_) and (*d*_22_, *o*_01_) are established through synapse dictionary *syn*. Synapses (*d*_12_, *o*_11_) and (*d*_12_, *o*_10_) establish channels between the input and the subsets having *x*_1_; synapses (*d*_22_, *o*_11_) and (*d*_22_, *o*_01_) establish channels between the input and the subsets having *x*_2_. At the same time, auxiliary neuron *σ*_2_ has one spike, rule *a* → *a* is applied and one spike is emitted to neuron *σ*_1_. The system after step three is shown in Fig 15.

**Fig 16:**
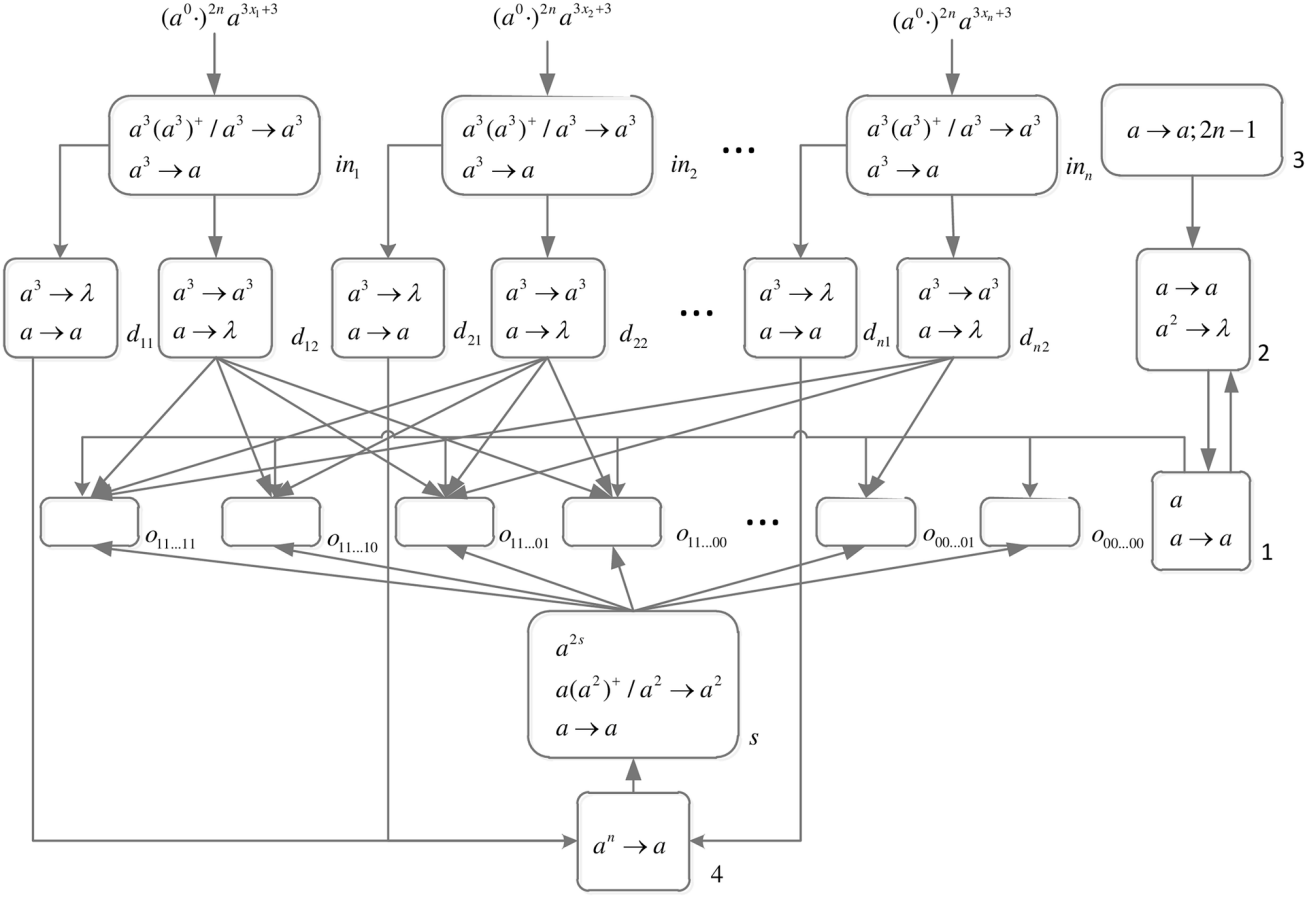
The system Π_*n*_ after step 2*n* − 1.

Similar process repeats. At step 2*n* − 1, 2^*n*^ neurons labeled *o*_*t*_1_*t*_2_…*t*_*n*__(*t*_1_, *t*_2_, …, *t*_*n*_ = 1, 2, …, *n*) are generated. The system after step 2*n* − 1 is shown in Fig 16.

**Fig 17:**
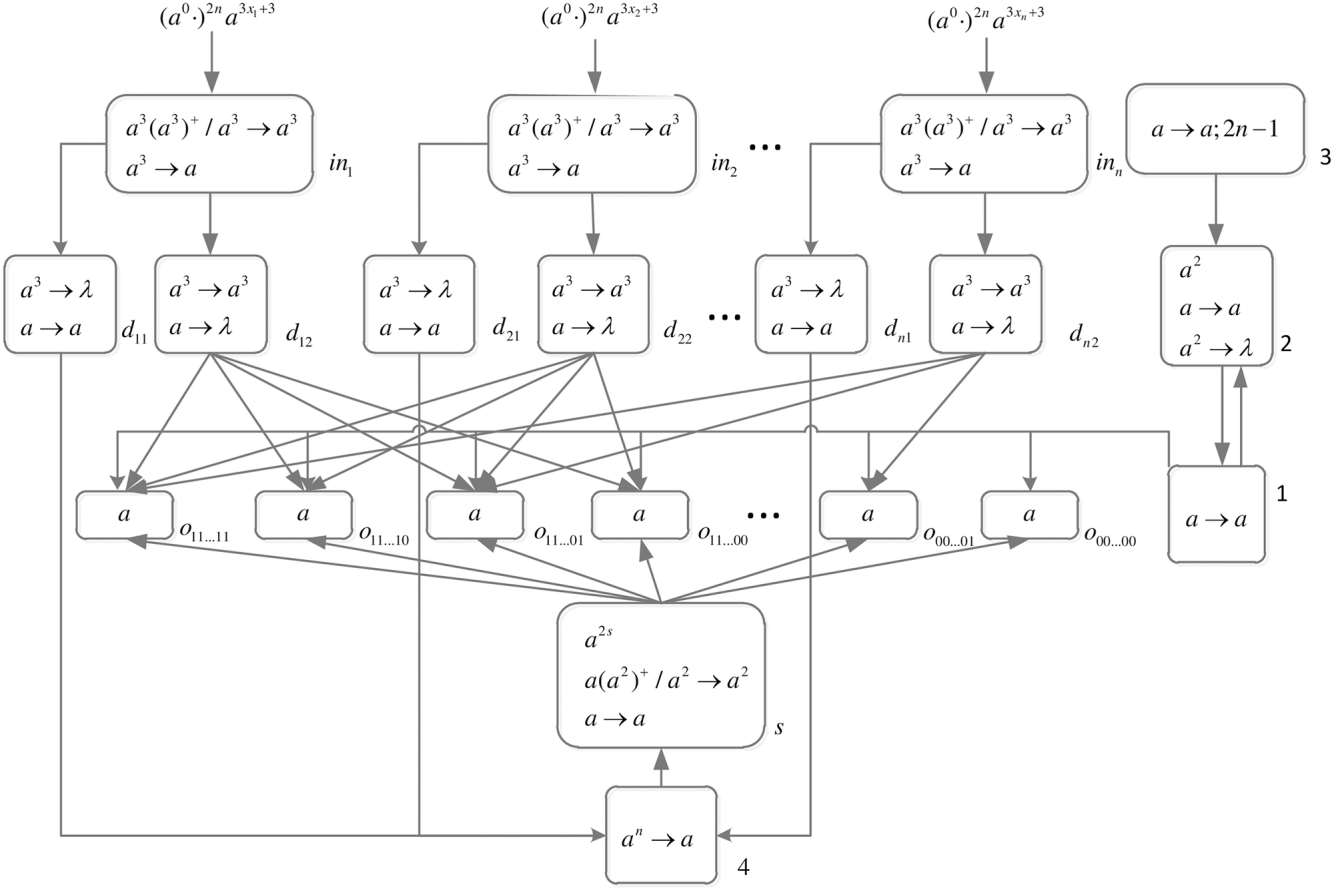
The system Π_*n*_ after step 2*n*.

At step 2*n*, each neuron *σ*_*o*_*t*_1_*t*_2_…*t*_*n*___ receives one spike emitted from neuron *σ*_1_ which will be deleted at the next step by the forgetting rule [*a* → λ]_*o*_*t*_1_*t*_2_…*t*_*n*___. Neuron *σ*_2_ receives two spikes (One is emitted from neuron *σ*_1_, and another one is emitted from neuron *σ*_3_.), the forgetting rule *a*^2^ → λ is applied at step 2*n* + 1, and no spike will be emitted to neuron *σ*_1_ later. The system after step 2*n* is shown in Fig 17.

*Input Stage*: At step 2*n* + 1, *x*_1_, *x*_2_, …, *x*_*n*_ enter the system through input neurons *σ*_*in*_*i*__(*i* = 1, 2, …, *n*). 3*x*_1_ + 3 spikes (*a*^3*x*_1_+3^) enter neuron *σ*_*in*_1__; 3*x*_2_ + 3 spikes (*a*^3*x*_2_+3^) enter neuron *σ*_*in*_2__;… 3*x*_*n*_ + 3 spikes (*a*^3*x*_*n*_+3^) enter neuron *σ*_*in*_*n*__.

At step 2*n* + 2, the firing rule *a*^3^(*a*^3^)^+^/*a*^3^ → *a*^3^ is applied, and three spikes are replicated and are emitted to neurons *σ*_*d*_*i*1__ and *σ*_*d*_*i*2__. Spikes in neuron *σ*_*d*_*i*1__ are forgotten, and spikes in neuron *σ*_*d*_*i*2__ are emitted to these *σ*_*o*_*t*_1_*t*_2_…,*t*_*n*___ having synapses going from neuron *σ*_*d*_*i*2__ to them (These neurons represent the subsets having the integer *x*_*i*_.) at step 2*n* + 3. This process repeats until only 3 spikes are in neuron *σ*_*in*_*i*__.

At step 2*n* + *x*_*i*_ + 2, the firing rule *a*^3^ → *a* is applied, and one spike is replicated and is emitted to neurons *σ*_*d*_*i*1__ and *σ*_*d*_*i*2__.

At step 2*n* + *x*_*i*_ + 3, the spike in neuron *σ*_*d*_*i*1__ is emitted to neuron *σ*_4_ showing that all spikes in neuron *σ*_*in*_*i*__ have been passed to neurons *σ*_*o*_*t*_1_*t*_2_…,*t*_*n*___ having synapses going from neuron *σ*_*d*_*i*2__ to them. The spike in neuron *σ*_*d*_*i*2__ is forgotten. Up to this step, 3*x*_*i*_ spikes are emitted to neurons *σ*_*o*_*t*_1_*t*_2_…,*t*_*n*___ which represent the subsets having the integer *x*_*i*_.

When all input spikes in neurons *σ*_*in*_*i*__ are passed to neurons *σ*_*o*_*t*_1_*t*_2_…*t*_*n*___ at step 2*n* + *x*_*max*_ + 3(*x*_*max*_ represents the maximum integer of all *n* integers.), neuron *σ*_4_ receives *n* spikes, and one spike is emitted to neuron *σ*_*s*_ at step 2*n* + *x*_*max*_ + 4. Up to this step, the number of spikes in neurons *σ*_*o*_*t*_1_*t*_2_…,*t*_*n*___ is $3\sum_{b\in B}b$.

*Checking Stage*: At step 2*n* + *x*_*max*_ + 5, 2*s* + 1 spikes are in neuron *σ*_*s*_, the firing rule *a*(*a*^2^)^ + ^/*a*^2^ → *a*^2^ is applied, two spikes are emitted to neurons *σ*_*o*_*t*_1_*t*_2_…*t*_*n*___. This process lasts for *S* circles.

At step 2*n* + *x*_*max*_ + *s* + 4, only one spike is in neuron *s*, and this spike is emitted to neurons *σ*_*o*_*t*_1_*t*_2_…*t*_*n*___.

There are three rule execution situations in neurons *σ*_*o*_*t*_1_*t*_2_…*t*_*n*___.

1. $\sum_{b\in B}b=S$
$3\sum_{b\in B}b$ spikes are in neuron *σ*_*o*_*t*_1_*t*_2_…*t*_*n*___ initially. 2 spikes are emitted to this neuron from neuron *σ*_*S*_, then forgetting rule *a*^2^(*a*^3^)^+^/*a*^5^ → λ can be applied with 5 spikes consumed. The number of spikes decreases to $3\left(\sum_{b\in B}b-1\right)$. This process repeats *S* times, and all spikes in neuron *σ*_*o*_*t*_1_*t*_2_…*t*_*n*___ are consumed. At this step, the last one spike is emitted to this neuron from neuron *σ*_*S*_, forgetting rule *a* → λ can be applied to consume this spike.
2. $\sum_{b\in B}b<S$
$3\sum_{b\in B}b$ spikes are in neuron *σ*_*o*_*t*_1_*t*_2_…*t*_*n*___ initially. 2 spikes are emitted to this neuron from neuron *σ*_*S*_, then forgetting rule *a*^2^(*a*^3^)^+^/*a*^5^ → λ can be applied with 5 spikes consumed. The number of spikes decreases to $3\left(\sum_{b\in B}b-1\right)$. This process repeats $\sum_{b\in B}b$ times, and all spikes in neuron *σ*_*o*_*t*_1_*t*_2_…*t*_*n*___ are consumed. At this step, two spikes are emitted to this neuron from neuron *σ*_*S*_, and neuron dissolution rule [*a*^2^]_*o*_*t*_1_*t*_2_…*t*_*n*___ → *δ*(*t*_1_ *t*_2_…*t*_*n*_ = 1, 2, …, *n*) is applied to dissolve this neuron.
3. $\sum_{b\in B}b>S$
$3\sum_{b\in B}b$ spikes are in neuron *σ*_*o*_*t*_1_*t*_2_…*t*_*n*___ initially. 2 spikes are emitted to this neuron from neuron *σ*_*S*_, then forgetting rule *a*^2^(*a*^3^)^+^/*a*^5^ → λ can be applied with 5 spikes consumed. The number of spikes decreases to $3\left(\sum_{b\in B}b-1\right)$. This process repeats *S* times, and $3\left(\sum_{b\in B}b-S\right)$ spikes are remaining. At the next step, the last one spike in neuron *σ*_*S*_ is emitted to this neuron, and dissolution rule [*a*(*a*^3^)^+^]_*o*_*t*_1_*t*_2_…*t*_*n*___ → *δ*(*t*_1_ *t*_2_…*t*_*n*_ = 1, 2, …, *n*) is applied to dissolve this neuron.

If some neurons *σ*_*o*_*t*_1_*t*_2_…*t*_*n*___ are still in the system after step 2*n* + *x*_*max*_ + *s* + 5, the labels of these neurons *σ*_*o*_*t*_1_*t*_2_…*t*_*n*___ are all solutions to this Subset Sum problem.

It can be seen that any *Subset* *Sum* *problem* (*n*) can be solved in linear time, and all solutions can be obtained through this system.

Some steps comparison results between our solution and other solutions, which use the non-deterministic method to solve the NP-complete problems, are shown in Table 2, where, *k* means that all *x*_1_, …, *x*_*n*_, *S* can be transformed into *k*-bit binary numbers.

**Table 2:** Steps comparison results of some uniform solutions to Subset Sum problem.

solution	step complexity (steps)	determinism or nondeterminism
SN P systems [19]	3*k* + 2	nondeterminism
SN P systems [20]	$3\Sigma_{i=1}^{n}x_{i}+6$	nondeterminism
SNPSP systems [22]	$2\Sigma_{i=1}^{n}x_{i}+6$	nondeterminism
time-free SN P systems [23]	$3\Sigma_{i=1}^{n}x_{i}+2$	nondeterminism
**DDSN P systems**	2*n* + *x*_*max*_ + *s* + 5	determinism

The conventional methods use the nondeterminism of SN P systems to solve Subset Sum problem, which means that whether a random combination of integers is one of the solutions or not can be checked by one computational process. These SN P systems can only judge whether a certain subset is the answer or not, but cannot search all solution space to judge whether a Subset Sum problem has solutions. Even if we let all combinations be traversed artificially to determine whether a Subset Sum problem has solutions or not, (2^*n*^ − 1)-times computations should be processed. Although the time complexity of each computation is a constant, the whole time complexity cannot be a polynomial of *n*. The proposed DDSN P system can solve the Subset Sum problem in a linear time, which improves the computational efficiency.

Considering a Subset Sum problem *Subset Sum problem (4)*: *X* = {1, 2, 3, 4}, *S* = 5, the DDSN P system Π_4_ is used to solve it. After 22 computational steps, neurons *σ*_*o*_0110__ and *σ*_*o*_1001__ are remaining which shows that {2, 3} and {1, 4} are all solutions to this Subset Sum problem. Methods proposed in [19, 20, 23] need 165 steps, 330 steps and 270 steps, respectively to judge this problem.

**Fig 18:**
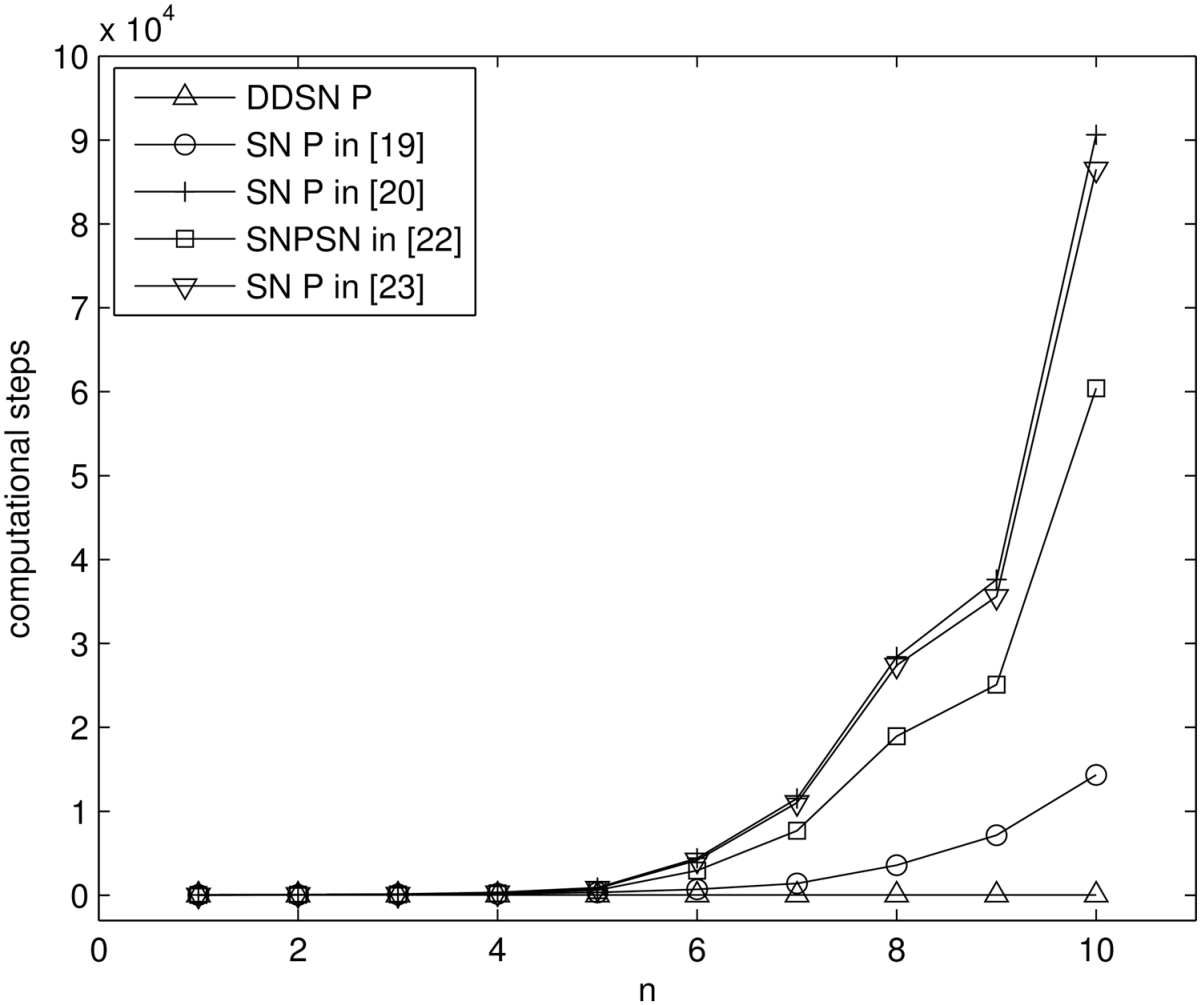
The computational steps of five types of SN P system solving Subset Sum problem.

A series of Subset Sum problems: *X* = {1, 2, …, *n*}, *S* = 5 are solved using the five systems in Table 2 and the computational steps of these systems are shown in Fig 18 by MATLAB R2014a. The computational steps of DDSN P system are much fewer, especially when the problem size is larger.

## 4 Conclusions

The new mechanism called neuron dissolution is introduced into the framework of SN P systems in this work. By this mechanism, redundant neurons can be dissolved immediately. The computational resources can be saved, which means more work can be done using the same resources, or the same work can be done using less resources. We also proved that this new variant of SN P system can obtain all solutions to NP-complete problems (Invalid solutions are eliminated by neuron dissolution.), such as SAT problem and the Subset Sum problem, in linear time, which enhances the application fields of SN P systems such as the register allocation problem.

This work provides a new thought of storing information in SN P systems, which can be used to store other information. The dissolution rule can be used to many situations to decrease the space complexity of a SN P system. This variant of SN P system can be used to solve other NP-complete problems and application problems. It is also an attractive direction to introduce other biological phenomena into SN P systems to reduce computational resources and enhance computational space efficiency.
